# Detergent Choice Shapes the Solution Structures of Photosystems I and II: Implications for Crystallization and High-Resolution Studies

**DOI:** 10.1021/acs.jpcb.5c00767

**Published:** 2025-08-08

**Authors:** M. Golub, J. Boyka, J. Gätcke, O. Hart, S. Haupt, D. C. F. Wieland, C. E. Blanchet, A. Zouni, J. Pieper

**Affiliations:** † Institute of Physics, 226264 University of Tartu, Wilhelm Ostwald str. 1, 50411 Tartu, Estonia; ‡ Department of Biology, 9373 Humboldt-Universität zu Berlin, 10099 Berlin, Germany; § Institute for Materials Research, Department for Metallic Biomaterials, 28338 Helmholtz Zentrum Hereon, Max-Planck-Straße 1, 21502 Geesthacht, Germany; ∥ 128803 European Molecular Biology Laboratory, Hamburg Outstation, Notkestrasse 85, 22603 Hamburg, Germany

## Abstract

Photosystems I (PSI) and II (PSII) are pigment–protein complexes that perform the light-driven charge separation necessary to convert solar energy into a biochemically usable form in a fundamental process called photosynthesis. Small-angle X-ray scattering provides unique structural insights into PSI and PSII in solution under near-physiological conditions. Here, we study the solubilization of PSI and PSII with different detergents, the octaethylene glycol monododecyl ether (C_12_E_8_) and the most commonly used n-dodecyl-β-D-maltoside (DDM). It is noteworthy that the volume of the C_12_E_8_ detergent belt is more compact for PSI and PSII than for DDM. Furthermore, circular dichroism measurements were used to detect thermal destabilization in protein solutions containing C_12_E_8_. The impacts of the size, number, mobility, and stabilization of the C_12_E_8_ molecules in the PSII complex solution before crystallization and after detergent extraction in the crystal are discussed in terms of obtaining an improved X-ray structure.

## Graphical abstract

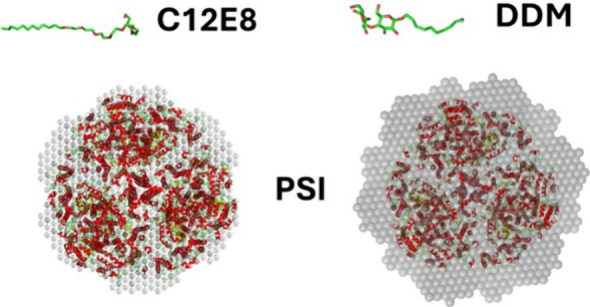

## Introduction

Photosynthesis is a vital biological process that produces oxygen, impacts the composition of our atmosphere, and forms energy-rich carbohydrates.^1^ The transformation of solar energy into chemical energy (photosynthesis) involves two membrane-enclosed pigment–protein complexes, Photosystem I (PSI) and Photosystem II (PSII), found in higher plants, algae, and cyanobacteria. PSII, a light-driven water plastoquinone oxidoreductase, extracts electrons from water molecules.^2–4^ PSI is responsible for the light-triggered electron transfer from reduced plastocyanin or cytochrome *c*_6_ to ferredoxin.

While these two integral protein complexes are of particular interest in photosynthesis research and biotechnology,^5–11^ their structural analysis is challenging, due to their extensive hydrophobic surface area, which, although crucial for stabilizing the protein within the native lipid bilayer, renders them insoluble in water. A detergent is required to extract the protein from the membrane, leading to the formation of a detergent belt around the hydrophobic surface. The resulting protein-detergent complex (PDC) is soluble in water due to its polar exterior.^12–14^ Among the variety of detergents accessible, n-dodecyl-β-D-maltoside (DDM) is widely used for most successful crystallization trials due to its nonionic and mild behavior.^15–19^ In our PSII X-ray structure, a total of 25 endogenous lipid molecules were identified in each monomer, including seven (33%) monogalactosyl diacylglycerol (MGDG) molecules and five (24%) digalactosyl diacylglycerol (DGDG) molecules, four (19%) sulfoquinovosyl diacylglycerol (SQDG) molecules and five (24%) phosphatidyl glycerol (PG) molecules (Table S1).^20,21^ This lipid composition is comparable to that of the thylakoid membrane.^22^

In an attempt to avoid unhinging of structurally relevant lipids from PSII, DDM was replaced by the nonionic detergent, octaethylene glycol monododecyl ether (C_12_E_8_), which has no structural similarities with galactolipids.^23^ However, using C_12_E_8_ resulted in the formation of type II dioxygen-active PSII crystals, as was previously demonstrated with DDM-PSII crystals.^20,23^ In this type of crystals, the space between protein units (Figure S1a) is usually large enough to accommodate the original detergent belt at the expense of stable protein contacts, impending the formation of high-quality crystals for diffraction experiments.^23–25^ Our recently developed postcrystallization protocol for C_12_E_8_ PSII crystals has revealed the extraction of intermolecular water and a depletion of detergent within the type II crystal.^23,25^ Consequently, a conversion into crystal type I is observed (s. Figure S1b). In type I crystals, in which membrane protein layers with protein contacts are stacked between transmembrane regions, detergent belts are absent. Therefore, type I crystals reflect a more native-like protein structure that most closely resembles the protein structure in the membrane.^26,27^ As a result of converting crystals from type II to I,^23^ the arrangement of PSII dimers into row-like structures was similar to that found in native thylakoid membranes of cyanobacteria.^23,28^ Thus, a new route to high-resolution PSII XFEL structure at physiological temperatures of at least 2.0 Å was found.^24,25,29^ The precise properties that the detergent in the PDC crystal must possess to convert a type II crystal into a type I crystal remain to be elucidated. A recent cryo-EM structure of the DDM PSII from was solved at 1.71 Å resolution^21^ and this technique allows for visualization of detergent belts in 3D reconstructions.^30^ However, the structure of a membrane protein in a buffer solution can differ from its crystalline form^13,31–35^ as well as from its low temperature cryo-EM structures, especially considering that the flexibility of PDCs drastically increases with temperature.^36,37^

Therefore, we chose small-angle X-ray scattering (SAXS)^38–41^ for the analysis of isolated membrane proteins under near-physiological conditions in solution, providing insights into the overall shape and size of PDC’s.^40^ In a more advanced approach, detergent molecules within PDCs^31–35^ or parts of a protein complex,^42^ can be selectively hidden using small angle neutron scattering (SANS) with contrast variation^38,43^ and specifically labeled detergent molecules.^44,45^ The aim of the present SAXS study is to investigate the effects of detergents on the crystallization process and the subsequent postcrystallization procedure within PDC solutions. Our measurements with PSI and PSII samples in both DDM and C_12_E_8_ detergents under near-physiological conditions revealed solution structures. In addition, the thermal stability of the two protein complexes in the various detergents was analyzed by circular dichroism (CD) spectroscopy. The results demonstrate the impact of detergents on the solution structures of PSI and PSII, with respect to the properties of the detergent shells surrounding the proteins. Finally, we consider the postcrystallization processes depending on the detergent in the PDC crystals, which led to a high-resolution PSII X-ray protein structure.^24,29^

## Materials and Methods

### PSI and PSII Preparation

PSI and PSII purification using either DDM or C_12_E_8_ is described in detail in the Supporting Information (SI). We verified the presence of all protein subunits in PSI and PSII (SDS-PAGE see SI, Figure S2 and MALDI-ToF see SI, Figure S3 and Table S2), purity, activity and homogeneity (BN-PAGE (see SI, Figure S2), size exclusion chromatography (see SI, Figure S4), DLS (see SI, p. S10)) of our protein samples by biochemical and biophysical analyses.

Precrystallized PSI was washed twice in salt-less buffer containing either 0.02% DDM or 0.013% C_12_E_8_ and then dissolved in the SAXS measurement buffer (either 25 mM Tricin, pH8; 200 mM MgSO_4_; 0.02% DDM or 5 mM MES, pH6; 30 mM MgSO_4_; 0.013% C_12_E_8_). PSII was diluted directly in the SAXS measurement buffer (either 0.1 M PIPES, pH7; 5 mM CaCl_2_; 0.03% DDM or 20 mM MES, pH6; 0.5 M Betain; 10 mM CaCl_2_; 0.02% C_12_E_8_). To ensure a complete buffer exchange, the PSI and PSII samples were washed three to four times in the measurement buffer in a centrifugal concentrator (Vivaspin 20, 100 kDa MWCO, Sartorius) and were adjusted to 0.47 mM chlorophyll concentration for PSII and 0.56 mM chlorophyll concentration for PSI prior to SAXS measurements. The homogeneity of PSI and PSII is verified by Pd < 15% of DLS measurements, resulting in 9.4 and 8.9 nm for 0.5 g/L PSI as well as 10.4 and 8.5 nm for 0.5 mM PSII in DDM and C_12_E_8_, respectively. The O_2_ consumption rates for DDM PSI and C_12_E_8_ PSI samples were measured at −1662 and 2150 μmol O_2_/(mg Chla · h) respectively, and the DDM PSII and C_12_E_8_ PSII samples showed O_2_ evolution rates of 1100 and 1600 μmol O_2_/(mg Chla · h), respectively.

### SAXS Experiments

Synchrotron SAXS experiments were conducted at the EMBL-P12 BioSAXS beamline of the PETRA III storage ring in DESY, Hamburg,^46^ using a continuous-flow batch mode and an automated robotic sample changer.^47^ Samples were loaded in 30 μL volumes at a controlled temperature of 20 °C and exposed to the X-ray beam. During this process, each image was captured over 0.1 s. This setup facilitated a total collection of 40 2D images per sample using a Pilatus 6 M detector. The continuous flow of the sample through the beam path significantly reduced radiation damage, enhancing the stability of the samples throughout the exposure. Subsequently, these 2D images were radially averaged to generate 1D scattering profiles. Before the final averaging step to produce these profiles, careful inspection was carried out to remove any images showing signs of radiation damage or artifacts such as detergent bubbles that could compromise the measurements. The scattering data from the corresponding buffer solutions containing detergents were subtracted from the sample scattering data to produce the final SAXS profiles (see SI, Figure S8). The setup employed a wavelength of 0.0124 Å and positioned the detector at a distance of 3 m from the sample, enabling measurements across a *Q* range from 0.0024 to 0.73 Å^–1^.

### SAXS Data Analysis

A model-independent approach to analyzing small-angle scattering data uses the Guinier approximation^48^

$$I(Q)=I(0)\exp\left(-Q^{2}\frac{R_{g}^{2}}{3}\right)$$ 1

where *R*_g_ is the radius of gyration, *I*(0) represents the forward scattering, a measure of the total scattering power independent of shape. *Q* is the scalar value of the scattering vector given by the formula:

$$Q=\frac{4\pi }{\lambda_{0}}\sin(\theta )$$ 2

where λ_0_ is the X-ray wavelength and θ is the scattering angle.

This approximation is applicable for monodisperse particles in dilute solutions within a small range of *Q* values where *QR*_g_ < 1.3.^48^

Since the concentrations of the protein-detergent complexes in solution were not precisely known, we used Primus software^40^ and got the molecular weights (*M*_w_) according to concentration-independent methods.^41,49,50^

The scattering correlates with a single particle averaged over all orientations in solutions where macromolecular particles are monodisperse. The relationship between the scattering intensity and a particle’s individual properties is outlined as follows:

$$I(Q)=4\pi \int_{0}^{D_{\max}}P(R)\frac{\sin(QR)}{QR}dR$$ 3

*P*(*R*) is the pair distance distribution function, which is nonzero from 0 to *D*_max_. *D*_max_ corresponds to the maximum interparticle distance.

The *P*(*R*) function and the maximum particle dimension *D*_max_ can be determined using the Inverse Fourier transform (IFT) method employing the software routine GNOM.^51^ For the IFT analysis, we used the limited *Q*-range up to 0.1 Å^–1^.

Further data analysis was carried out using dedicated software packages developed by Svergun and co-workers.^41,52,53^ In the present work, we present DAMMIF sphere structures reconstructed from the *P*(*R*) function corresponding to the overall shape of the complexes.^52,53^ This study’s structural models were derived using the DAMMIF average over 20 iterations. For each iteration, it is taken into account that both PSI and PSII proteins have oblate shapes. In addition, the PSI structure requires a *P*_3_ symmetry, while the PSII structure shows a *P*_2_ symmetry, which were imposed on the respective structures in the modeling process.

Another approach to the data analysis involves the usage of the MPBuilder plugin to the PYMOL program (PyMOL, Version 0.99)^54^ which is capable of forming a protein-detergent complex based on the known crystal structures of PSI (pdb 6trd^35^) and PSII (pdb 5kaf^55^) with DDM or C_12_E_8_. The pdb codes of the detergent were taken from Charmm-Gui web service,^56^ which is a known online tool for molecular dynamic simulations. The pdb structures of the complexes delivered by the MPBuilder plugin were taken as the basis for further modifications in the Pymol program. Finally, the Crysol^57^ and PEPSI-SAXS^58^ software have been applied to fit the experimental SAXS data according to estimated model structures.

### Circular Dichroism Spectroscopy Experiments

We performed CD spectroscopy with a J-815 JASCO spectropolarimeter from the University Potsdam (group of physical biochemistry) for the determination of the melting temperature *T*_m_ of the unfolding reaction. The temperature in the 1 mm quartz cuvette (polarimetric, 110–1-P-40, Hellma) was set by a Peltier thermostated cell holder (PTC-423S, JASCO). CD spectra were recorded in the range of 200–250 nm at a scanning speed of 50 nm/min, a bandwidth of 0.5 or 1 nm and accumulation of 3 scans. The temperature was increased from 20 to 95 °C and for the PSI samples with C_12_E_8_ up to 80 °C. Temperature-dependent unfolding of the α-helical components of the protein was normalized to the mean value of around −20 mdeg for PSI and to −33.5 mdeg for PSII.

The buffers used for CD measurements are either G60 (5 mM MES; pH6; 60 mM MgSO_4_; 0.02% DDM) and storage buffer (5 mM MES pH 6; 30 mM MgSO_4_; 0.013% C_12_E_8_) for PSI and for PSII the buffer is composed of 20 mM MES pH6 and 10 mM CaCl_2_ with either 0.02% DDM or 0.013% C_12_E_8_.

For measurement and export of the data the Jasco Spectra Manager 2 software was used.

By assuming a two-state reversible equilibrium process between the native (*F*) and denaturated states (*U*) the fraction of denaturated protein, *f*_U_, at temperature, *T*, was determined according to the following eq 4):

$$Uf_{U}=\frac{\Theta_{T,222}-\Theta_{F}}{\Theta_{U}-\Theta_{F}}$$ 4

where Θ_*T,*222_ is the measured ellipticity at 222 nm and temperature *T*. Θ_F_ and Θ_U_ are the ellipticities at 222 nm of a totally folded and unfolded states measured at 25 and 95 °C in case of DDM and 80 °C for samples with C_12_E_8_, respectively.

The denaturation curve was analyzed with the nonlinear Boltzmann eq 5) of OriginPro, Version 2023 (OriginLab Corporation, Northampton, MA, USA):

$$y=A_{2}+\frac{A_{1}-A_{2}}{1+e^{(x-x_{0})/dx}}$$ 5

to derive the *T*_m_ (= *x*_0_), with the initial and final CD plateau signals are denoted as *A*_1_ (= Θ_F_) and *A*_2_ (= Θ_U_) respectively, and dx is the slope of the curve.

## Results and Discussion

### Model-Independent Structural Analysis

The results detailed below provide comprehensive structural insights from the SAXS analysis of isolated and highly purified PSI and PSII solubilized using DDM and C_12_E_8_ detergents, respectively. Initial observations from the SAXS curves indicate substantial differences in the scattering profiles between the complexes formed with DDM and with C_12_E_8_, as depicted by the red and black curves in Figure 1, respectively. Notably, the SAXS data for PSI and PSII in the presence of DDM reveal a distinct peak around a *Q* of 0.13–0.15 Å^–1^, which is virtually absent in the curves of samples with C_12_E_8_. The latter feature can be attributed to free detergent micelles, which are taken into account in detailed models discussed further below. Another important observation is the steeper decay slope of the SAXS curves for DDM-containing samples within the *Q* range of 0.03 to 0.1 Å^–1^ compared to that measured for C_12_E_8_ (see Figure 1A,B).

**Figure 1:**
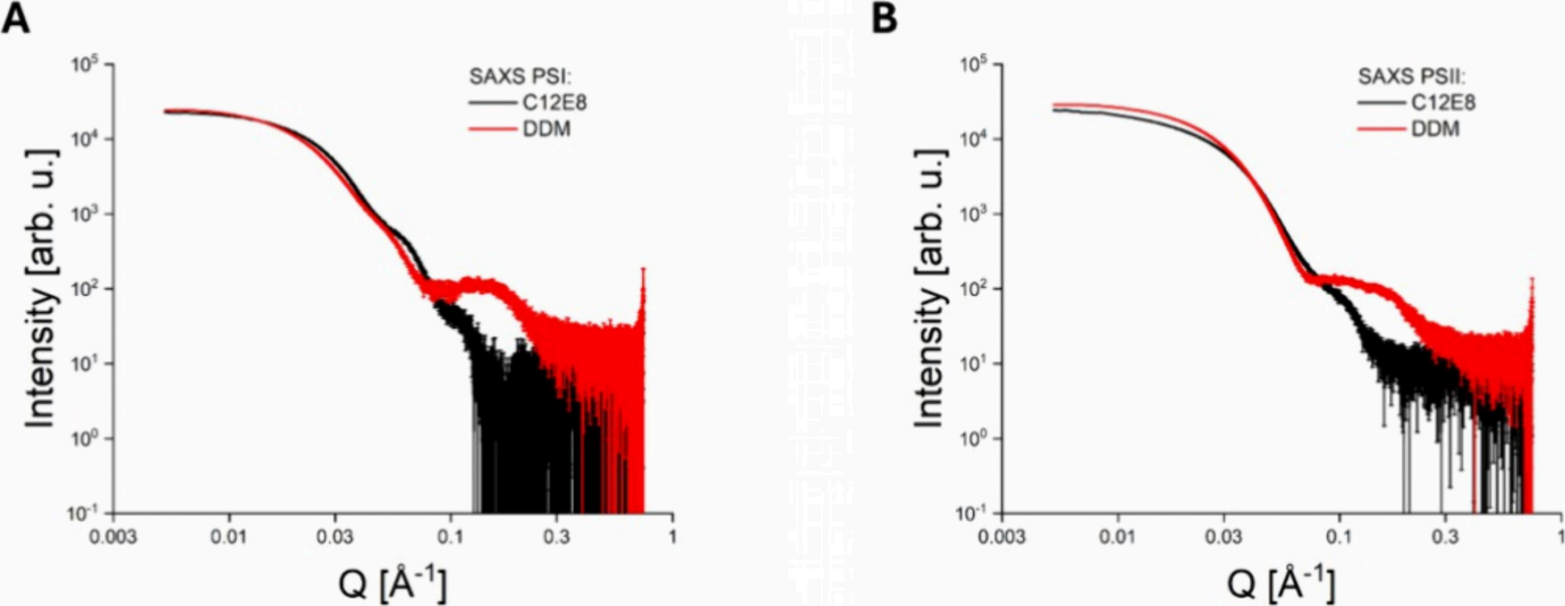
Panel A: SAXS data of PSI in solution with C_12_E_8_ detergent and DDM detergent, shown as black and red curves, respectively. Panel B: SAXS data of PSII in solution with C_12_E_8_ detergent and DDM detergent, shown as black and red curves, respectively.

We first start with a model-independent analysis limited to the *Q*-range <0.1 Å^–1^, where the influence of effects stemming from free detergent micelles should be largely negligible. In the case of the PSI samples, the model-independent analysis indicates that both *R*_g_ and *D*_max_ are larger when DDM is used compared to C_12_E_8_. Specifically, the *R*_g_ for PSI solubilized with DDM was measured at approximately 80 ± 2 Å, while it was about 73 ± 2 Å for the C_12_E_8_ PSI complex. For context, the theoretical *R*_g_ for the crystal structure of the DDM PSI trimer is approximately 68.2 Å. Similarly, *D*_max_ values were 270 ± 10 and 237 ± 10 Å for the DDM PSI and C_12_E_8_ PSI complexes, respectively (for more details, see Table 1 and Top panel of Figure 2). The same trend is observed in molecular weight (*M*_w_) calculations, where the *M*_w_ of the C_12_E_8_-PSI complex is about 1150 kDa, compared to about 1500 kDa for the DDM-PSI complex.

**Table 1:** Model-Independent Analysis

	*M*_w_ (kDa)	*R*_g_ (Å)	*D*_max_ (Å)
PSI crystal (6trd)	1027 ^35^	68.2	206.5
DDM PSI	1500 ± 200	80 ± 2	270 ± 10
C_12_E_8_PSI	1150 ± 200	73 ± 2	237 ± 10
PSII crystal (5kaf)	654 ^55^	62	187
DDM PSII	940 ± 200	69 ± 2	230 ± 10
C_12_E_8_PSII	840 ± 200	70 ± 2	210 ± 10

**Figure 2:**
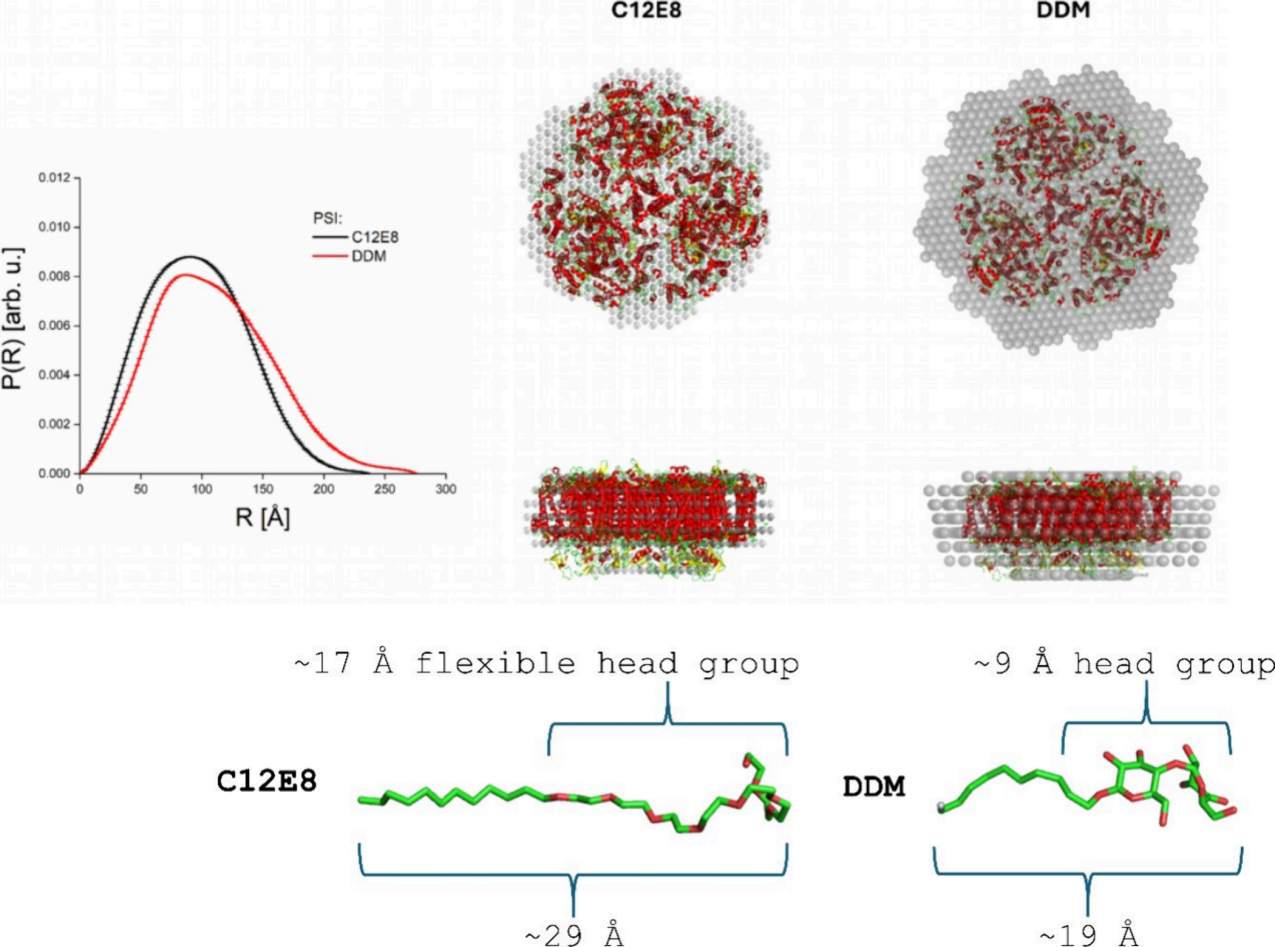
Top panel: DAMMIF analysis of DDM PSI and C_12_E_8_ PSI SAXS curves. On the left side, the *P*(*R*) functions calculated by the Gnom program for C_12_E_8_ PSI and DDM PSI SAXS curves are shown as black and red solid lines, respectively. On the right side, we present the sphere structures produced by DAMMIF fitting. As the reference, the sphere structures are compared to the known crystal structure of PSI (pdb 6trd^35^) shown as red-yellow cartoons. Bottom panel: Schematic representation of C_12_E_8_ and DDM detergent molecules with their approximate dimensions. The structures of the detergent molecules are shown as green and red sticks representing carbon and oxygen atoms, respectively. The pdb codes of the detergents were taken from Charmm-Gui web service.^56^

The model-independent analysis yields very similar values of *R*_g_ and *D*_max_ for both DDM PSII and C_12_E_8_ PSII SAXS curves (*R*_g_ = 69 ± 2 Å and *D*_max_ ≈ 220 ± 10 Å, see Table 1). This fits well to the recently published cryo-EM DDM PSII structure^21^ reporting a *D*_max_ of 238 Å (see SI, Figure S9). *M*_w_ calculations suggest a notable difference in molecular mass: the DDM PSII complex is approximately 939.9 kDa, whereas the *M*_w_ of the C_12_E_8_ PSII complex is only 843.3 kDa. These preliminary findings suggest that the PDCs formed with DDM are significantly larger than those with C_12_E_8_, despite DDM’s smaller molecular size (for more details, see bottom panel of Figure 2).

### Ab Initio Structural Reconstitution

The top panel of Figure 2 shows a DAMMIF structural model of PSI solubilized in DDM and C_12_E_8_, respectively, derived from SAXS data. On the left, the *P*(*R*) functions for PSI in both DDM (red curve) and C_12_E_8_ (black curve) are displayed. The right side shows sphere models fitted via DAMMIF alongside the known crystal structure of PSI (pdb 6trd), colored in red and yellow. Consistent with the model-independent analysis, the DAMMIF modeling reveals variations in the size and shape of the PDCs depending on the detergent used, impacting their interaction and stabilization of PSI. Notably, the sphere models suggest cylindrical shapes with the same length but different radii, with the DDM complex displaying a noticeably larger radius. This effect on the cylinder radius suggests a more voluminous DDM detergent belt covering PSI’s hydrophobic surface than the C_12_E_8_ detergent belt. Interestingly, despite the smaller detergent belt of C_12_E_8_, the C_12_E_8_ PSI complex remains stable, suggesting that its stabilization requires less detergent, which may be more efficient in shielding PSI’s hydrophobic surfaces without the bulk of a larger detergent envelope.

Contrary to expectations that a smaller DDM detergent molecule would result in smaller detergent belts, our results indicate that DDM might instead form larger structures.

The DAMMIF analysis of PSII solubilized in DDM and C_12_E_8_ detergents (see Figure 3) show a similar pattern as observed for PSI. The modeled sphere structures of the DDM PSII and C_12_E_8_ PSII complexes resemble elliptical cylinders, sharing similar lengths and major radii.

**Figure 3:**
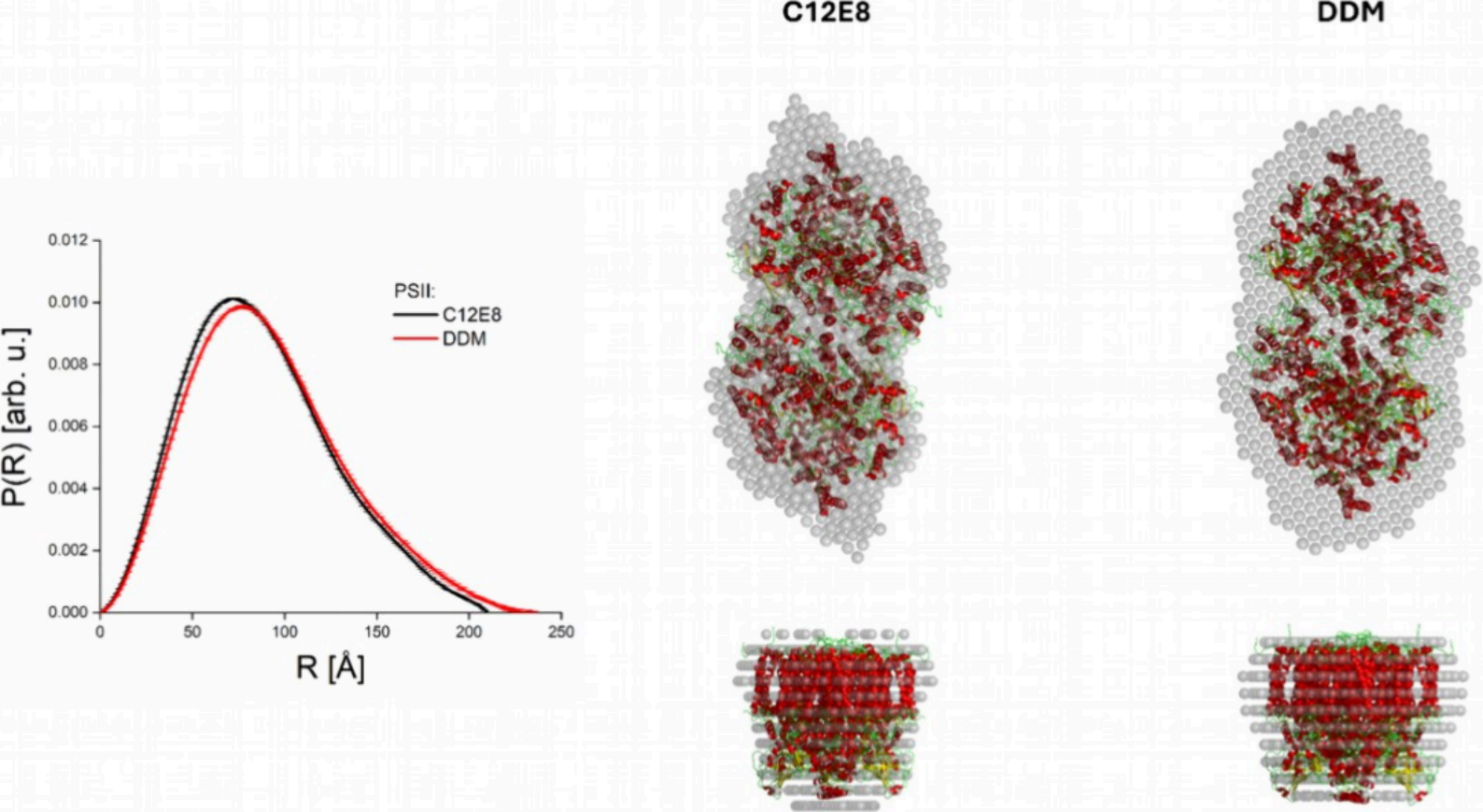
DAMMIF analysis of DDM PSII and C_12_E_8_ PSII SAXS curves. On the left side, the *P*(*R*) functions calculated by the Gnom program for C_12_E_8_ PSII and DDM PSII SAXS curves are shown as black and red solid lines, respectively. On the right side, we present the sphere structures produced by DAMMIF fitting. As the reference, the sphere structures are compared to the known crystal structure of PSII (pdb 5kaf^55^), shown as red-yellow cartoons.

However, the minor radius is notably larger in the DDM PSII complex due to the more voluminous DDM detergent belt, highlighting differences in how each detergent interacts with the protein complexes.

Based on the sphere structure of the C_12_E_8_ PSII complex, one can speculate that the C_12_E_8_ detergent belt structure is very compact on the elongated sides of PSII.

Interestingly, both DDM PSII and C_12_E_8_ PSII crystallize in crystal form II using the salting-out method.^23^ Surprisingly, the different size and number of detergent molecules in the shells do not seem to play a significant role in the crystallization behavior of the two PDCs. Nevertheless, there is a significant difference in the resolution of the resulting PDC X-ray structures. While the DDM PSII X-ray structure is at 2.9 Å resolution, the X-ray structure of C_12_E_8_ PSII only reaches a resolution of 6.0 Å.^20,23^ This result can be explained by the higher flexibility of the C_12_E_8_ molecules in the detergent shell in the PSII crystal, which may lead to a weakening of the crystal contacts.^23^

Even more intriguing is the PDC SAXS structural data regarding postcrystallization treatment on the type II C_12_E_8_ PSII crystals. In this instance, a postcrystallization treatment with poly ethylene glycol monomethyl ether 5000 (PEG5000 MME) removes the water molecules and the detergent substantially, resulting in a repacking to type I crystals accompanied by a significant improvement in resolution.^24,25^ This treatment works well with C_12_E_8_ but not with DDM. Interestingly, the headgroup of C_12_E_8_ is itself a PEG-like molecule. Thus, we propose that PEG as precipitating agent not only influences the protein–protein interaction, but also the stability of the detergent belt. There are two aspects to be considered in the postcrystallization process of the C_12_E_8_ PSII crystals. First, the PEG increases the solubility of the detergent monomers in the aqueous phase, as suggested by the critical micelle concentration (CMC), which is slightly larger for C_12_E_8_ than for the DDM.^23^ Second, the addition of PEG to the crystals facilitates the disintegration of the detergent shell into monomers. It seems that PEG shows a greater stabilizing effect on the C_12_E_8_ molecules in aqueous phase than the DDM. The SAXS structures of C_12_E_8_ PSII in solution show a potentially slimmer and more flexible C_12_E_8_ shell, which is probably also present in the PSII crystals. Therefore, we hypothesize that the subsequent extraction with PEG leads to an easier removal of the C_12_E_8_ molecules from the shell in the PSII crystals than the larger, bulkier and hydrogen bond-forming headgroup in the DDM shell. This observation could improve our understanding of the mechanism of detergent extraction by postcrystallization treatment of protein crystals.^59^

### Structural Models of Detergent Belts

We now proceed to a reconstruction of the structures of PDCs including their detergent belts. Figure 4 illustrates our efforts using the MPBuilder plugin to estimate the structure of the C_12_E_8_ PSII complex (see details in the Materials and Methods section). The structures produced by MPBuilder were applied to fit the measured SAXS data using Crysol. The right panel of Figure 4 displays the best-fitting model (side and top views of the C_12_E_8_ PSII complex structure) achieving a fit with the minimal χ^2^ of 5.04. This complex is delineated by a belt comprising 196 detergent molecules, resulting in a molecular weight (*M*_w_) of approximately 127.4 kDa for the belt alone and 781.4 kDa for the entire complex, corroborating our *M*_w_ estimates from the Porod volume analysis detailed in Table 1.

**Figure 4:**
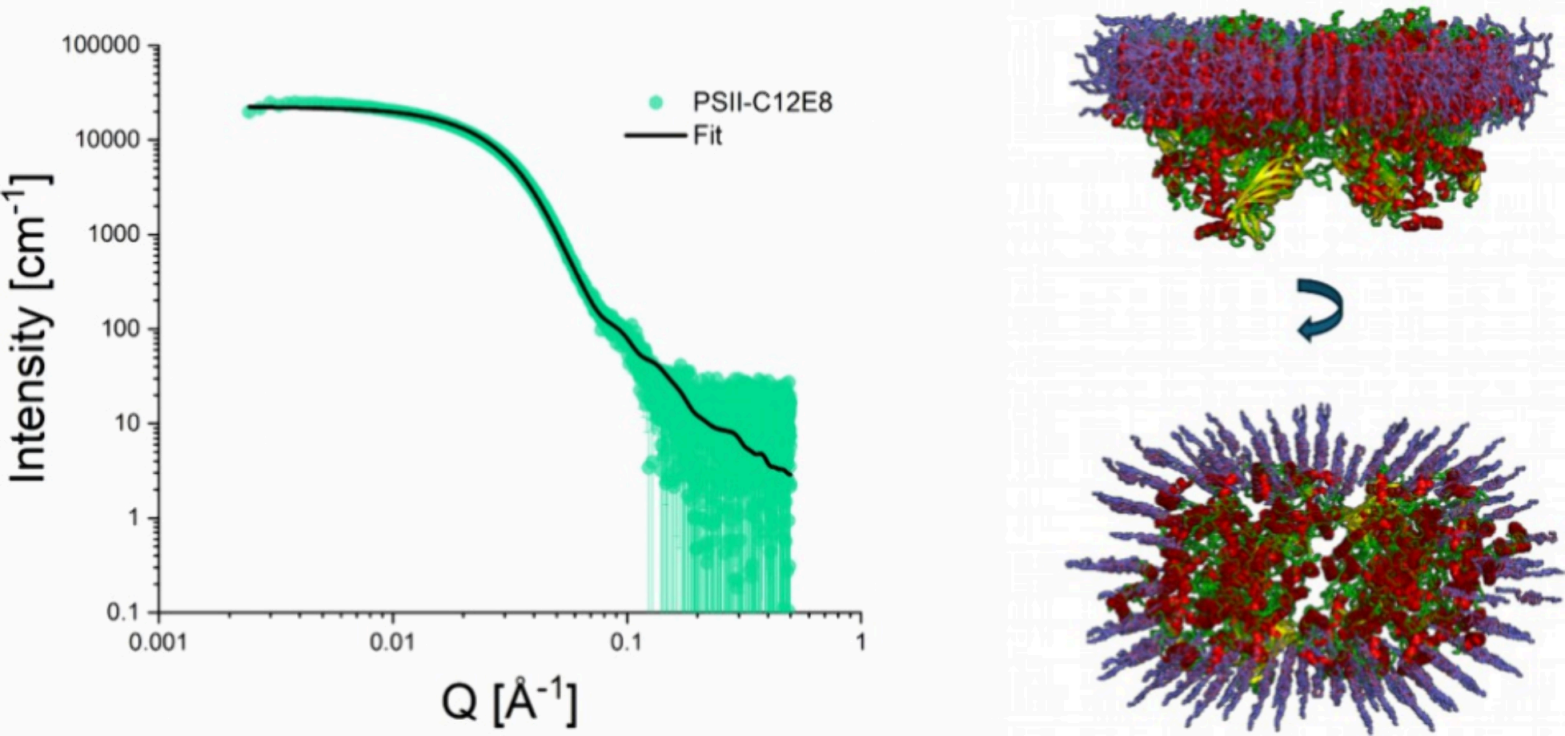
SAXS data analysis by the MPBuilder plugin^40^ (shown on the left) and the corresponding reconstitution of the C_12_E_8_ PSII complex structure (shown on the right). The red-yellow-green cartoons represent the known PSII crystal structure (pdb 5kaf^55^), and the violet sticks – C_12_E_8_ detergent molecules.

Figure 5 shows the best attempt to apply MPBuilder to fit the C_12_E_8_ PSI SAXS curve. The complex model contains 133 C_12_E_8_ detergent molecules (Table 3), which give rise to a *M*_w_ of 63.9 kDa. The total *M*_w_ of the complex is about 1090.9 kDa, which is in line with our concentration-independent estimation of *M*_w_. One sees that the modeled curve has a prominent minimum at *Q* of 0.9, which is not present in the measured curve. That leads to a relatively high χ^2^ of 6.11. Thus, one may speculate that C_12_E_8_ does not form a very structured monolayer belt. As an explanation, we suggest that C_12_E_8_ remain in the elongated form suggested by the atomic structure (see Figure 2) and used for modeling here. Rather, the hydrophobic tail is highly flexible resulting in a very heterogeneous composition of the detergent belt, so that the volume of the actual belt is reduced in line with the sphere structure calculated using Dammif. Therefore, it is not possible to reproduce the C_12_E_8_ belt in atomic details at this point. Nevertheless, we can estimate a number of detergent molecules according to the *M*_w_ and prove the stability of the C_12_E_8_ PSI complex, which is the essential message for further development of the postcrystallization protocols for PDCs.

**Figure 5:**
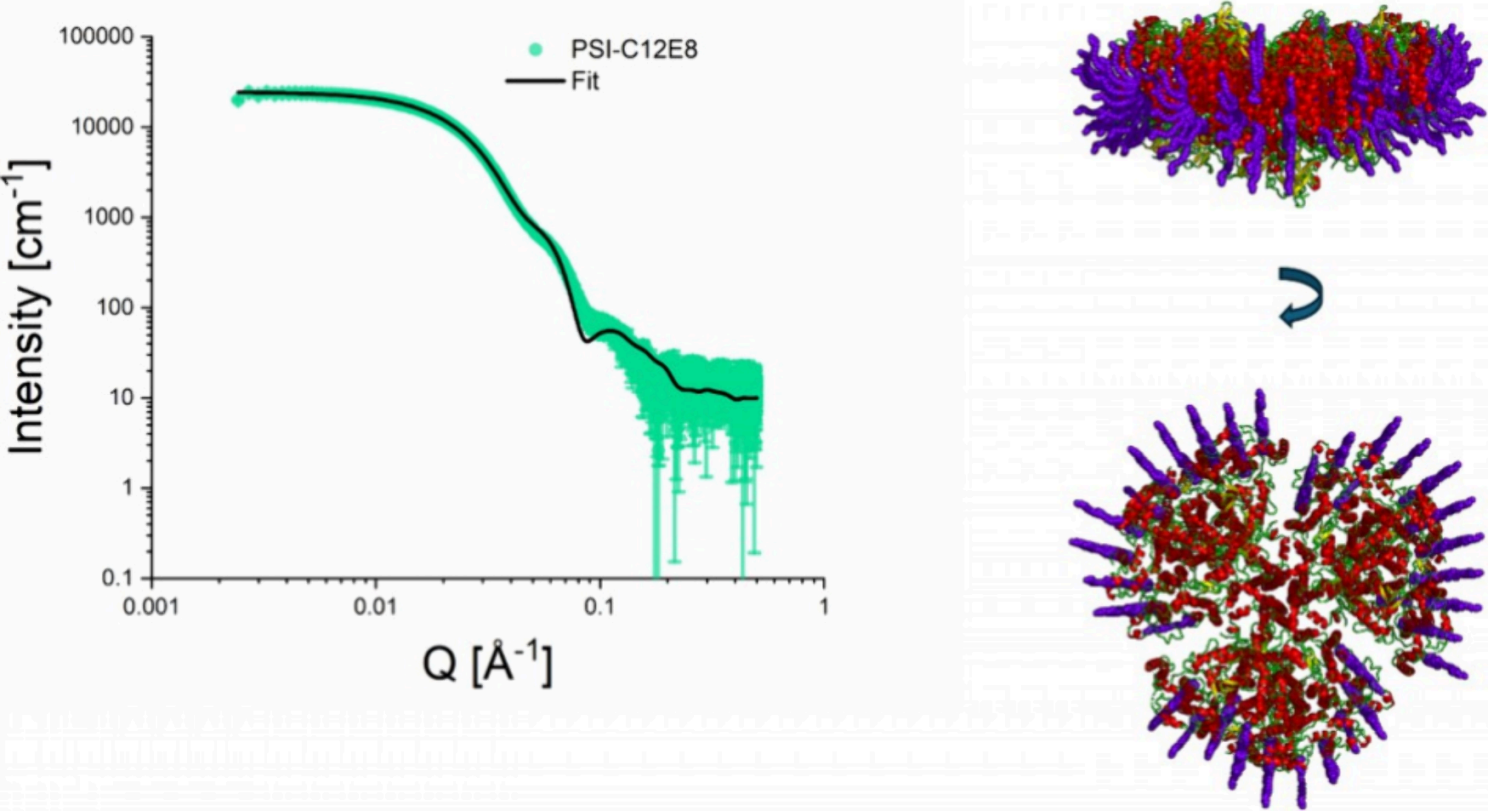
SAXS data analysis by the MPBuilder plugin^40^ (shown on the left) and the corresponding reconstitution of C_12_E_8_ PSI complex structure (shown on the right). The red-yellow-green cartoons represent the known PSI crystal structure (pdb 6trd^35^) and the violet sticks – C_12_E_8_ detergent molecules.

### Free Detergent Micelles

As mentioned above, the SAXS data of complexes containing DDM exhibited an additional feature tentatively associated with free detergent micelles. This requires further effort in data analysis and fitting. Figure 6 shows a fit the DDM-PSII SAXS curve. Notably, we obtained an excellent fit using MPBuilder, but only for the *Q* range up to 0.08–0.1 Å^–1^, since no structural model of the DDM PSII complex is able to reproduce the strong peak with a maximum at *Q* of 0.13–0.15 Å^–1^. We have observed a similar feature before in the X-ray scattering profile of the DDM-PSII complex.^12^ Our conclusion was that this may be a result of additional scattering contributions from free detergent micelles present in the solution with the DDM PSII complex. Using the same modeling approach, we obtained an excellent fit of the DDM PSII SAXS curve as a sum of the scattering profile of the DDM PSII complex (see the gray dashed line in Figure 6) and the micelle scattering profile taken from our previous study (see the gray dotted line in Figure 6). The right panel of Figure 6 represents the final model of the DDM PSII complex, which includes 250 DDM detergent molecules with a *M*_w_ of the DDM belt equal to 127.9 kDa. Therefore, the formation of a detergent belt around PSII may require almost one-quarter more DDM than C_12_E_8_ molecules. Assuming that the detergent belt in the DDM crystal would also be larger than in the C_12_E_8_ crystal and due to the more hydrophobic properties of DDM, the removal of the DDM shell in the PSII crystal may be more difficult or even impossible.

**Figure 6:**
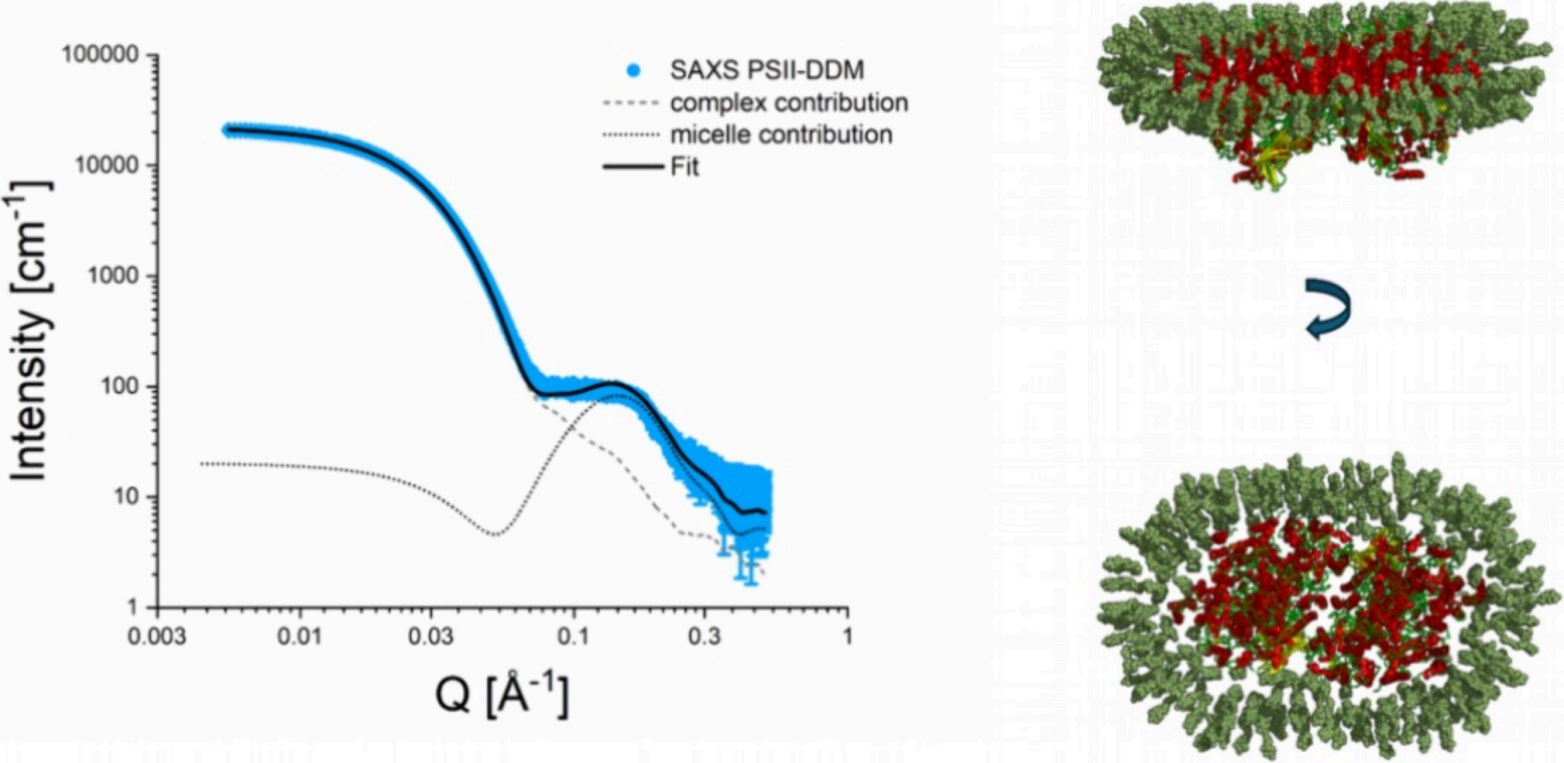
SAXS data analysis (shown on the left) and the corresponding reconstitution of the DDM PSII complex structure (shown on the right). The Fit curve is obtained as a sum of two scattering contributions: DDM PSII complex, reconstituted by the MPBuild plugin, and a model micelle shown as gray dashed and dot lines, respectively. The red-yellow-green cartoons represent the known PSII crystal structure (pdb 5kaf^55^) and the green sticks – DDM detergent molecules.

In total, the *M*_w_ of the DDM PSII complex is 781.9 kDa, which is smaller than the estimate from the concentration-independent analysis of *M*_w_; however, the mismatch can be understood due to the presence of the additional micelles. Thus, we can roughly calculate the *M*_w_ of the micelles as 150 kDa, which is the difference between the *M*_w_ obtained from the concentration-independent method and the *M*_w_ of the DDM PSII complex reconstituted by MPBuilder.

The aggregation numbers (*m*) of C_12_E_8_ (*m* = 120)^60^ and DDM (*m* = 100–150)^34,60,61^ are similar and for DDM this corresponds to a *M*_w_ of 34–76 kDa.^62,63^ For further calculations we use a *M*_w_ of 53 kDa for DDM.^64^ This finding suggests that the profile’s scattering component is different from DDM micelles. To verify this hypothesis, we independently measured the scattering profile of DDM micelles (see Figure 7). We tried improving the automatic buffer subtraction, assuming that the DDM micelle concentration might differ in the buffer and the solution with the DDM PSII complex.

**Figure 7:**
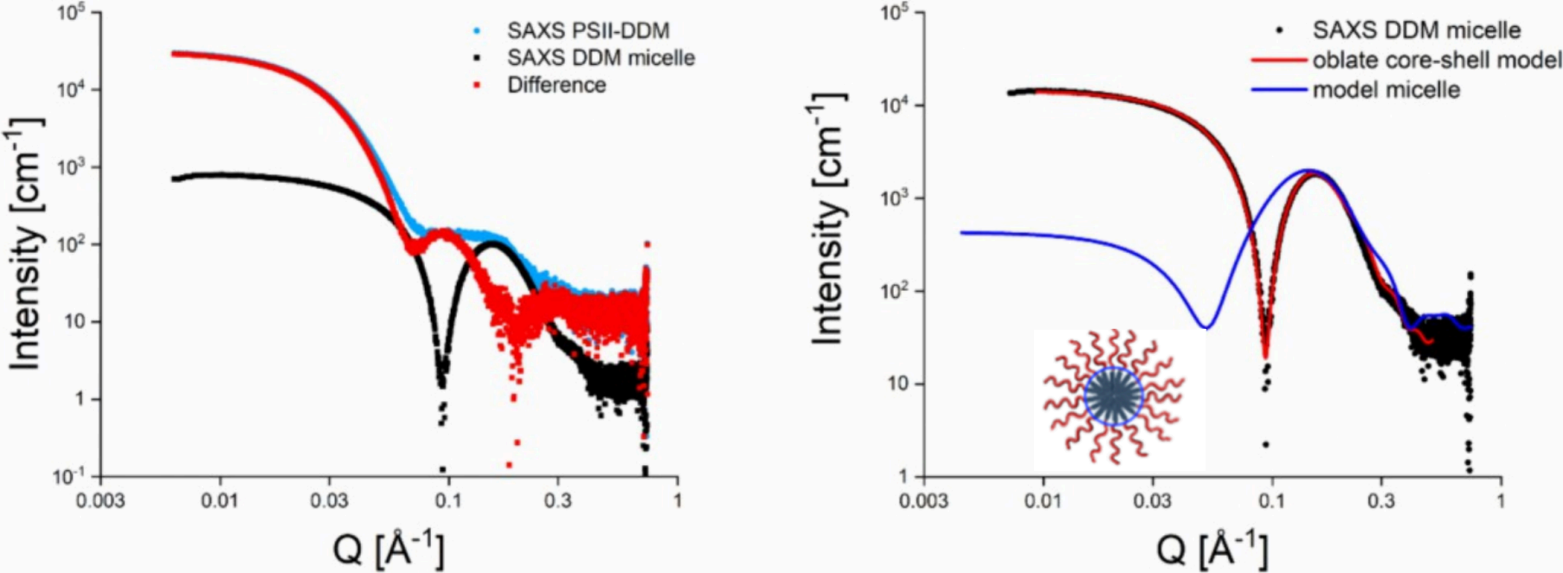
Left panel: Measured SAXS curves of the DDM PSII sample (blue points) and DDM micelle (black points). The red points represent the subtraction difference between the two SAXS curves. Right panel: Measured SAXS curve of the DDM micelle (black points). The red line reveals the fitting curve of the DDM micelle SAXS curve by the model of the oblate core–shell ellipsoid (see the fitting parameters in Table 1). The blue line shows the model SAXS curve for the micelle contribution used in the fit in Figure 6 (see the model parameters in Table 2).

Despite our efforts, subtracting the measured scattering profile of the DDM micelle did not eliminate the peak at *Q* of 0.13–0.15 Å^–1^, as illustrated in the left panel of Figure 7. The resulting curve, highlighted with red points, unexpectedly showed a prominent peak at *Q* of 0.1 Å^–1^, which could not be replicated in our modeling efforts. Table 2 outlines the dimensions of the oblate core–shell ellipsoid model used to fit the DDM micelle’s SAXS profile (see the red line on the right panel of Figure 7), contrasting with the micelle profile depicted as a gray dotted line in Figure 6 or as a blue line on the right panel of Figure 7. The DDM SAXS curve fitting suggests the micelle has an elongated core–shell ellipsoid shape with a relatively thin shell of 7 Å compared to a major core radius of 29 Å. Meanwhile, the modeled profile suggests a more spherical micelle with a smaller hydrophobic core radius of 11 Å and a shell thickness more than twice as large at 25 Å. After subtracting the buffers containing only protein-free DDM micelles, a larger unknown micellar structure remains in the PDC solution. We hypothesize that those are protein-free lipid-detergent mixed micelles whose lipid-detergent composition is still unclear. In the near future, we will investigate the composition of the different lipid micelles in the DDM PSI and PSII solutions by native MS, LC-MS and also laser-induced liquid bead ion desorption (LILBID-MS).^65–67^ These micelles, of unknown structure, cannot be separated during concentration of the PDC by ultrafiltration with a 100 kDaA MWCO filter. It should be noted that micelles are in dynamic exchange with neighboring lipid-containing micelles throughout the purification process, and their composition changes.^68^ We hypothesize that the formed DDM micelles may not only interfere with the crystal growth of DDM PSI and DDM PSII crystals, but also increase the effective size of the detergent belt surrounding the transmembrane domain of the protein, making the crystal contacts sterically unfavorable.^68^ A quantification of the detergent and lipid concentrations in the PDC solution as a proportion of protein to PDC prior crystallization is a crucial prerequisite for an optimal growth of PDC crystals (see SI FTIR section). We used Fourier-transform infrared spectroscopy (FTIR) to determine the detergent concentration by using the protein (at 2870/cm) and PDC (at 2855/cm) peak. These measurements^68^ could be performed in-house and were precise for the detergent concentration in the buffers but for PDC solutions the detergent concentrations showed different results. Moreover, MALDI-ToF^64^ for determining the detergent content is planned in the near future.

**Table 2:** Micelle Profile

oblate core–shell ellipsoid		
	DDM micelle measured (red line on the right panel in Figure 7)	model micelle (blue line on the right panel in Figure 7)
major radius core [Å]	29	11
minor radius core [Å]	14	11
shell thickness [Å]	7	25

The same peak feature observed in the scattering profile of the DDM PSI sample mirrors that observed in the DDM PSII analysis, necessitating the inclusion of an additional scattering contribution from a model micelle. By integrating the scattering contributions of the DDM PSI complex (indicated by the gray dashed line in Figure 8) with that of the model micelle, we achieved an excellent fit of the DDM PSI SAXS curve. The scattering profile for the model micelle was kept consistent with that used for the DDM PSII sample, differing only in a scaling prefactor.

**Figure 8:**
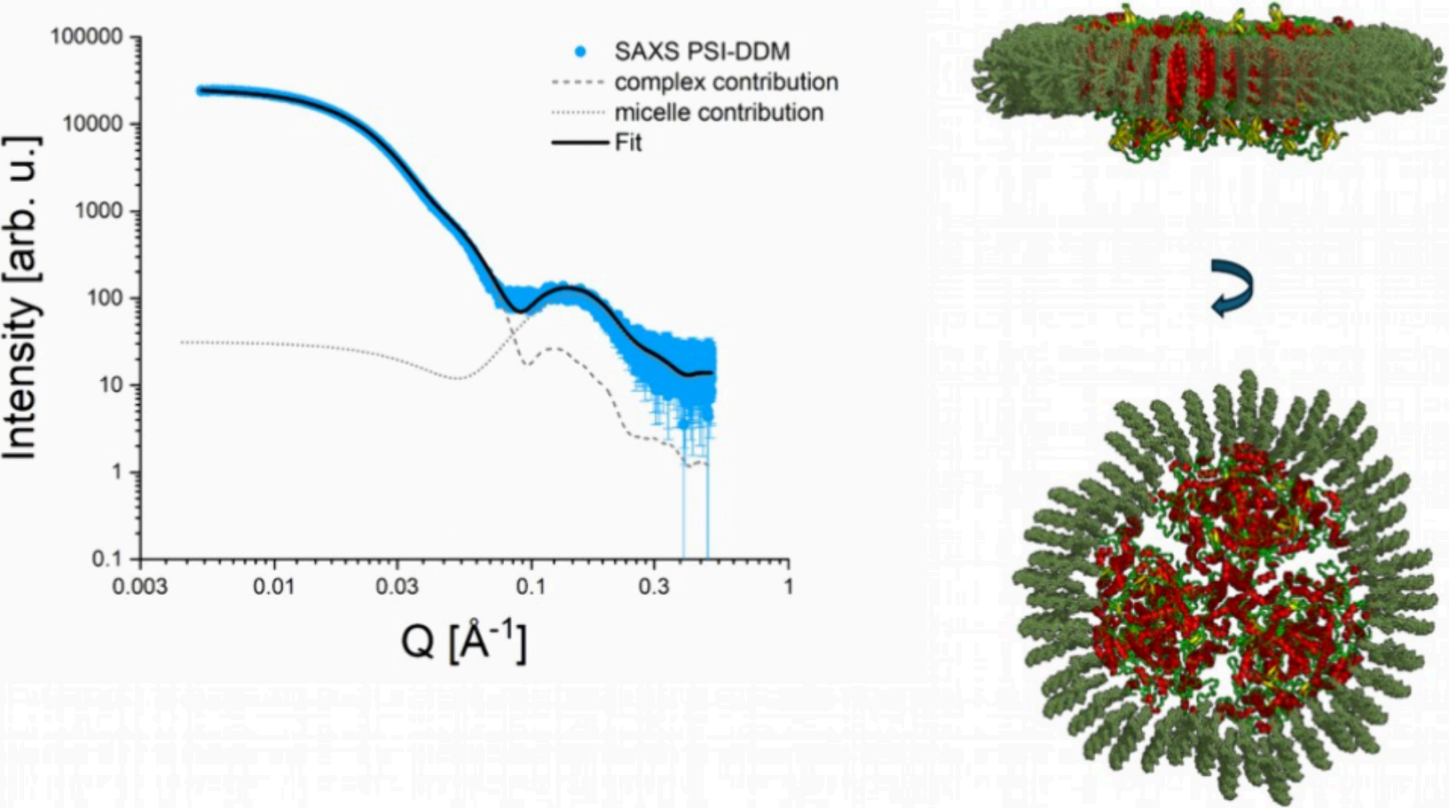
SAXS data analysis (shown on the left) and the corresponding reconstitution of the DDM PSI complex structure (shown on the right). The Fit curve is obtained as a sum of two scattering contributions: DDM PSI complex, reconstituted by the MPBuild plugin, and a model micelle shown as gray dashed and dot lines, respectively. The red-yellow-green cartoons represent the known PSI crystal structure (pdb 6trd^35^), and the green sticks – DDM detergent molecules.

Interestingly, no additional peak could be observed for the C_12_E_8_ PSI and PSII samples after buffer subtraction (see Figure S8 in the SI). Even though we cannot exclude the presence of unknown micelles of the same size as the detergent micelles present in the buffer but there are definitively no larger unknown micelles present in the C_12_E_8_ samples as we detected for DDM. In contrast to the DDM PSI and PSII solutions, the extraction of the PDC with C_12_E_8_ may rarely result in the formation of larger lipid-C_12_E_8_ clusters. The SAXS data have shown that the detergent belt of C_12_E_8_ around the proteins is smaller. The optimal model structure for the DDM PSI complex, which best fits the SAXS curve, incorporates 693 DDM detergent molecules forming a detergent belt that covers the hydrophobic surface of PSI. This structure aligns well with the spherical structure generated by DAMMIF for the DDM PSI SAXS data set. The molecular weight of this DDM belt is 354.5 kDa, contributing to a total molecular weight of 1381.5 kDa for the DDM PSI complex. This finding is consistent with the concentration-independent *M*_w_ analyses with the assumption of considering micelle contributions to the overall scattering profile.

Our result indicates that the number of detergent molecules for DDM compared to the C_12_E_8_ molecules in the detergent belt for PSI is about a factor of ∼5.2 larger than for PSII with ∼1.3. The new approach to analyze the SAXS data via MPBuilder^40^ revealed the detergent molecule numbers in the detergent belt of PSI and PSII shown in Table 3.

**Table 3:** Determined Number of Detergent Molecules Present in the Detergent Belt

	C_12_E_8_	DDM
PSI	133	693
PSII	196	250

### Implications of Lipid and Detergent Molecules in PDCs

In the framework of our long-term PSII structural research aiming at a better understanding of the function of lipids and detergents in PDC,^20,23,29^ we will discuss the influence of the two structurally different detergents in combination with the CMC and the aggregation number (*m*) on lipidation: (i) in the detergent PSII complex, (ii) on the surface of the protein and (iii) in the detergent-lipid envelope. Since both detergents have similar aggregation number, as mentioned above, detergent micelles of similar size should form. In addition, both detergents have similar CMC values for C_12_E_8_ (0.09 mM)^69^ and DDM (0.17 mM),^34^ which are far exceeded during the protein purification process. Accordingly, we expect a similar behavior for both detergents with respect to protein lipidation. The following needs to be considered: (i) Due to the structural similarity of DDM to the galactolipid molecules (DGDG and MDGD), lipid exchange with DDM molecules occurs in the protein, which we identified in the DDM PSII X-ray structure.^20^ Since the C_12_E_8_ molecule has no structural similarity to the galactolipids, the exchange in PSII should be only partial, resulting in a more native PSII.^23^ However, endogenous PSII internal lipids were found to retain C_12_E_8_-PSII overall, while those at the periphery, including the monomer–monomer interface, were likely exchanged. Exceptions include two pairs of lipids specifically bound at both the monomer–monomer and dimer–dimer interfaces. (ii) However, two previously undetected lipids (MGDG 785 and 789) were identified on the surface of the protein, which were apparently retained during the extensive purification in C_12_E_8_. They are located at the interface between PSII dimers, which may be of physiological relevance for dimer–dimer interactions *in vivo*.^23,70^ We speculate that this may be due to an increased exchange of galactolipids from the protein surface with DDM molecules in the detergent shell of the PDC, possibly leading to the formation of larger mixed lipid-detergent micelles which are in equilibrium with other micelles in solution. (iii) Furthermore, we have no information on the number, composition and structure of the lipid detergent molecules in the detergent-lipid shell of the PDC.^71^ Remarkably, our SAXS data show that the detergent belt of C_12_E_8_ around the proteins is smaller. Therefore, we assume that the interactions between the lipids and the C_12_E_8_ molecules in the solution are weaker, so that possibly hardly larger lipid C_12_E_8_ clusters can be formed. The reason for this is still unclear. Since the aggregation numbers of both detergents are similar, the differences in the shell sizes cannot be explained by the interactions between the detergent molecules or by packing criteria according to Israelachvili et al.^72^ Possible reasons for this are differences in detergent-protein interactions and the resulting ability of detergents to displace lipids, which then penetrate the envelope and change its size. However, we can only speculate here.

Both detergents possess identical hydrophobic tails (C12) and the differences arise from the chemical structure and interaction potential of the respective head groups. C_12_E_8_ features a polyethylene glycol (PEG)-based headgroup composed of eight ethylene oxide units, which is highly flexible, extensively hydrated, and lacks functional moieties capable of forming strong or specific interactions with polar or charged amino acid residues on the protein surface. Consequently, the protein–detergent interface is governed predominantly by nonspecific hydrophobic interactions, resulting in a relatively weak and dynamic association. Thus, the endogenous lipids in PSII should have no structural or functional similarity to the galactolipid head groups prevalent in the thylakoid membrane.

By contrast, DDM contains a maltoside (disaccharide) headgroup bearing multiple hydroxyl groups, which can form specific hydrogen bonds with side chains of residues such as serine, threonine, and tyrosine, as well as backbone carbonyls. These interactions contribute to a more stable and specific detergent belt around the protein, thereby promoting stronger overall interactions and enhanced structural preservation. The sugar-based headgroup of DDM better mimics natural lipids, allowing for stronger interactions and potentially improving the retention of native lipids and the structural integrity of detergent-lipid micelles.

In summary, for a more accurate analysis, MD simulations and lipid analyses^70^ of the PDC in solution are required.

### Thermal Stability of PSI and PSII Measured by Circular Dichroism (CD) Spectroscopy

The stability of PSI and PSII solutions in DDM and C_12_E_8_ as well as their micelle mixtures was investigated as a function of temperature using CD spectroscopy (Figure 9A,B). The DDM purified PSI and PSII exhibit the highest measured stabilities of the two PDCs in solution with the phase transition temperature (*T*_m_) values of about 81 °C, respectively (see Table 4). In contrast, the stability of the purified C_12_E_8_ PSI sample decreases by about 15 °C compared to the DDM protein complexes, and the C_12_E_8_ PSII decreases even more dramatically by about 20 °C. Interestingly, both PSI and PSII samples with additional C_12_E_8_ show the lowest stability, regardless of whether PSI and PSII were purified with DDM or C_12_E_8_ (see Table 4).

**Figure 9:**
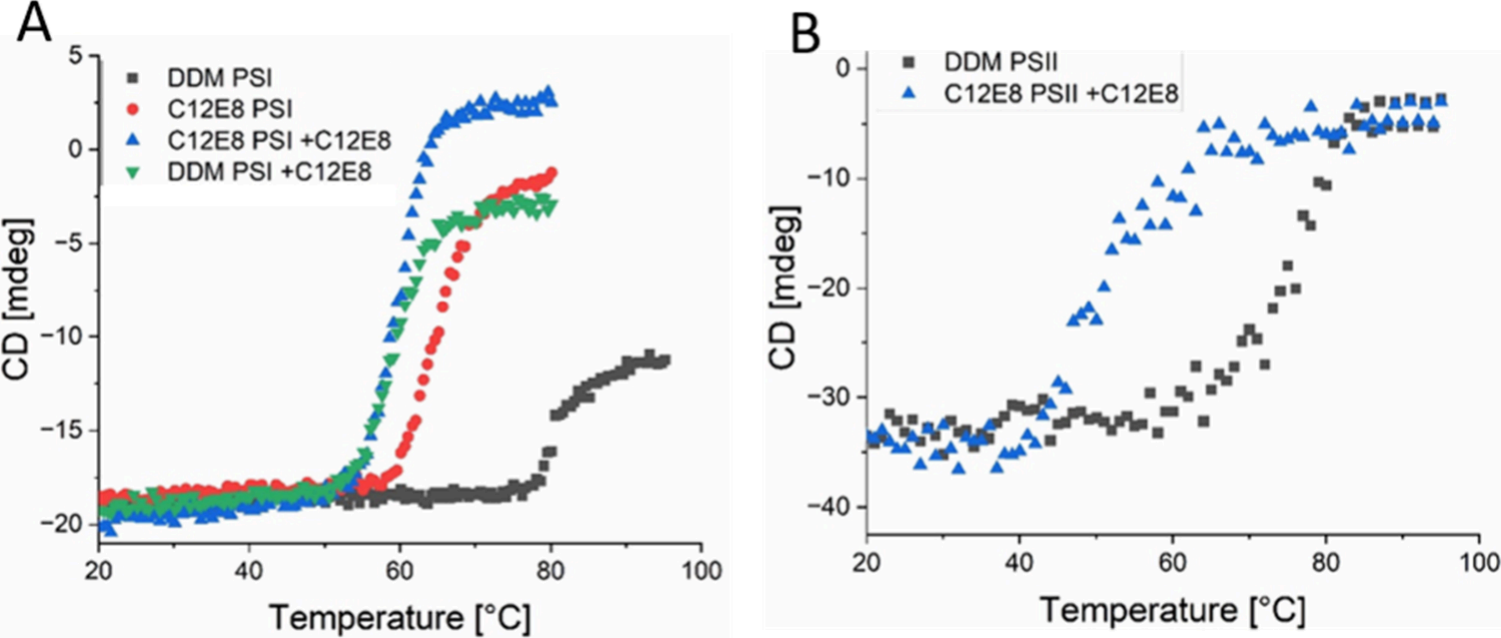
Denaturation of PSI and PSII induced by a temperature increase as observed with CD spectroscopy (*n* ≥ 3). (A) Fraction of unfolded PSI purified with either DDM (black squares) or C_12_E_8_ (red circles), C_12_E_8_ purified PSI with additional C_12_E_8_ (blue triangles) and DDM purified PSI with additional C_12_E_8_ added to the solution (green triangles). (B) Fraction of unfolded PSII purified with either DDM (black squares) or C_12_E_8_ purified PSII with additional C_12_E_8_ (blue triangles).

**Table 4:** Determined Phase Transition Temperature (*T*_m_) for PSI and PSII in Different Detergents and Their Mixed Micelles

	phase transition temperature (*T*_m_) [°C]	
detergents	PSI	PSII
DDM	81	80
C_12_E_8_	65	59^a^
DDM + C_12_E_8_	59	54.5^a^
C_12_E_8_ + C_12_E_8_	60	58

a Not shown in Figure 9.

The CD data clearly show that the presence of C_12_E_8_ drastically destabilizes the DDM shell in the PDC. This fits well with our SAXS results, which showed that the DDM shell of the protein complexes consists of much larger and more rigid molecules and should therefore, be more stable than the smaller and more flexible C_12_E_8_ detergent molecules in the shell around the PDC. Therefore, our CD data support the hypothesis that the flexible C_12_E_8_ molecules enveloping the protein complex are more likely to reflect the mobile native lipid molecules in the membrane.^28^

In addition, the *T*_m_ values indicate that the stability of the DDM protein complex decreases significantly with increasing C_12_E_8_ concentration (see Table 4). It is assumed that the added C_12_E_8_ molecules partially replace the DDM molecules in the detergent belt of the protein complex and form a mixed micelle with significantly reduced stability. Adding more C_12_E_8_ to the DDM protein complex reduces the stability to an almost pure C_12_E_8_ protein complex (see Table 4).

Moreover, we used CD spectroscopy to compare the stability of the C_12_E_8_ - DDM mixed micelles at the different PDCs (Table 4). This suggests that not only the C_12_E_8_ molecules in purified C_12_E_8_-PSII can be extracted during the postcrystallization process,^23^ but possibly also in a mixed micelle present in the crystal. This could be a great advantage, as the already very well purified and crystallizable DDM protein complexes are available in large quantities.^25,73^ This in turn would lead to a significantly improved X-ray structure of the PDC.^24,74^

## Conclusions

Our SAXS measurements confirm that both the C_12_E_8_ PSI and C_12_E_8_ PSII complexes are properly solubilized and remain stable over time.

Furthermore, the structural data of C_12_E_8_ PSII and C_12_E_8_ PSI showed that the C_12_E_8_ shell of PSII is 1.3 times and of PSI about 5 times smaller than the corresponding DDM shells. Therefore, we can conclude that due to the smaller and more flexible C_12_E_8_ molecules in the detergent belt in type II PSII crystals, removal of the detergent by postcrystallization treatment with PEG is feasible and results in a conversion to crystal form I.^23,29,59^ The native-like PSII arrangement in the crystals accompanied by the significant improvement of the XFEL structure of at least 2.0 Å resolution. This was the breakthrough for the investigation of the light-driven dynamic process of the water splitting reaction in PSII during the Kok cycle at the molecular level.^24,29,75^

Noticeably, we found that the DDM PSII crystals could not be induced to postcrystallization treatment because of their large and bulky shell which may limit natural protein–protein interactions in PSII that is typically observed in thylakoid membranes. Nevertheless, we cannot exclude the possibility of a conversion of the DDM PSII crystals of type II into I under other conditions.^23,59^

We hypothesize that postcrystallization treatment is also feasible on C_12_E_8_ PSI crystals, since the detergent shell is composed of significantly fewer and more flexible C_12_E_8_ molecules, which interact more weakly with the hydrophobic surface of the protein, thus result in a lower stability of the PDC as shown in our CD spectroscopy measurements. Similar to PSII, the detergent could also be removed, which would be accompanied by a type II to I conversion. Consequently, a more compact and native-like structure of PSI could be generated in the crystal,^76,77^ leading to a near-atomic resolution of a PSI-XFEL structure.^74,78^

In the DDM PSI and DDM PSII complexes in solution, larger micelles of unknown composition were detected by SAXS data. Remarkably, these mixed micelles could interfere with the crystallization of the PDC, resulting in poorer resolution of the protein structures.^68^ Interestingly, no mixed C_12_E_8_ micelles of the purified C_12_E_8_ protein complex could be detected by SAXS measurements. This might be an advantage for the crystallization of C_12_E_8_ protein complexes. Nevertheless, a tandem mass spectrometry-lipidomic analysis, as shown for the trimeric DDM PSI,^70^ should be performed for our PDC samples combined with a quantification of the detergent. A comprehensive analysis of the latter is planned for the near future.

We point out explicitly that delipidation can occur not only within the protein itself but also on its hydrophobic surface during the processes of solubilization and purification with detergents.^20,70,79^ This often resulted in the loss of the native conformation, or even of protein subunits.^80,81^ Novel alternative membrane mimetic systems (MMs)^82^ such as nanodiscs (NDs),^83^ styrene-maleic acid lipid particles (SMALPs),^70,84^ or peptide disks (PDs) have been developed to create a near-natural environment in the form of a lipid bilayer and preserve the natural conformation and function in solution. In addition, Brady et al. showed for a PSI-SMALP complex from (PSI-SMALP) that it exhibits significantly faster energy transfer and charge separation *in vitro* than detergent-isolated PSI complexes.^70^ ,^84^ This unique technique has already yielded numerous native cryo-EM protein structures.^85,86^ A copolymer of a linear α-olefin and maleic acid (αMAs) was recently shown to promote the formation of PSI-containing nanodiscs retaining a lipid ring and native-like activity^87^ providing new structural insights into more nativelike PSI and II complexes.

In summary, our results provide a deeper insight into how the size, type, shape and number of detergent molecules around the PSI and PSII detergent complex can influence the stability and thus the crystallization behavior. In particular, improved X-ray structures can be achieved by postcrystallization treatment of PDC crystals, which can lead to an in-depth knowledge of the structure–function relationships at the molecular level of the PDC.

## Supplementary Material



## Notes

Crystal packing type I and II (Figure S1), list of endogeneous lipid and detergent molecules in (Table S1), protein isolation procedure, sample purity (BN- and SDS-PAGE in Figure S2, MALDI-ToF spectra in Figure S3, subunits in Table S2, size exclusion chromatography in Figure S4), activity and DLS measurements of PSI and PSII samples, detergent determination (Figures S5 and S6), SAXS measurements of the buffer only (Figure S7) and the buffer subtraction of C_12_E_8_ PSI and PSII (Figure S8), and determination of the detergent belt of C_12_E_8_ PSII (Figure S9) of previously published Cryo-EM data (pdb 9evx, map emd_50019)^21^ (PDF)
