# Supplementary material for: Detergent Choice Shapes the Solution Structures of Photosystems I and II: Implications for Crystallization and High-Resolution Studies
Source: J Phys Chem B. 2025 Aug 8;129(33):8392–405. doi: 10.1021/acs.jpcb.5c00767 (PMC12376099; doi:10.1021/acs.jpcb.5c00767)
Supplement: Supplementary file 1 [file jp5c00767_si_001.pdf]

Supporting Information

Detergent choice shapes the solution structures of Photosystems I and II: implications for crystallization and high-resolution studies

M. Golub[a], J. Boyka[b], J. Gätcke[b], O. Hart[b], S. Haupt[b], D. C. F. Wieland[c], C. E. Blanchet[d], A. Zouni[b], and J. Pieper[a]*

[a] *Institute of Physics, University of Tartu, Wilhelm Ostwald str. 1, 50411 Tartu, Estonia*

[b] *Department of Biology, Humboldt-Universität zu Berlin, 10099 Berlin, Germany*

[c] *Helmholtz Zentrum Hereon, Institute for Materials Research, Department for Metallic Biomaterials, Max-Planck-Straße 1, 21502 Geesthacht, Germany*

[d] *European Molecular Biology Laboratory, Hamburg Outstation, Notkestrasse 85, Hamburg, 22603, Germany*

*Author to whom correspondence should be addressed:

Jörg Pieper

Institute of Physics
University of Tartu
W. Ostwald str. 1
50411 Tartu, Estonia

phone.: +(372) 737 4627
email: pieper@ut.ee

M. Golub[a], J. Boyka[b]: These authors contributed equally to this work.

Crystal packing type I and type II

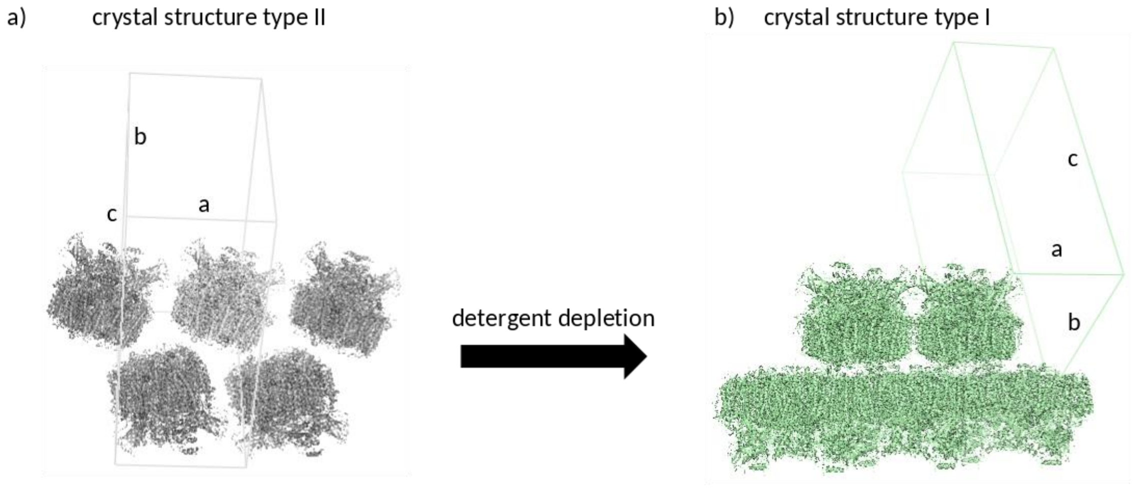

Figure S1: Depiction of PSII a) type II crystals (pdb 2axt[1], DDM) and b) type I crystals (pdb 7rf1[2], $C_{12}E_8$) together with the respective unit cell.

Table S1: Listed are the number of endogenous lipid and detergent molecules per monomer found in DDM and $C_{12}E_8$ PSII structures as well as the composition of the thylakoid membrane (Thylakoids) of *T. vestitus*; Abbrev. monomer 1 (m1), monomer 2 (m2) of PSII, monogalactosyl diacylglycerol (MGDG), digalactosyl diacylglycerol (DGDG), sulfoquinovosyl diacylglycerol (SQDG), phosphatidyl glycerol (PG).

**molecules**	DDM PSII pdb 9evx[3]	$C_{12}E_8$ PSII pdb 7rf1[2]		Thylakoids
		m1	m2	(*T. vestitus*)[4]
MDGD	7 (33 %)	9 (39 %)	6 (32 %)	~ 45 %
DGDG	5 (24 %)	4 (17 %)	5 (26 %)	~ 25 %
SQDG	4 (19 %)	5 (22 %)	3 (16 %)	~ 15-25 %
PG	5 (24 %)	5 (22 %)	5 (26 %)	~ 5-15 %
detergent (DDM)	7	-		-

Protein isolation

Cultivation of *T. vestitus* BP-1 and membrane protein extraction were performed as reported previously.[5] *T. vestitus* cells were grown in 30 l of modified Castenholz Medium D[6] (0.52 mM Ethylenediaminetetraacetic acid (EDTA); 1 mM KNO_3; 0.41 mM $MgSO_4$; 1.4 mM NaCl; 8 mM $NaNO_3$; 1 mM Na_2HPO_4; 0.027 mM Fe-EDTA; 6 mM $NaHCO_3$ und 0.35 mM $CaCl_2$ and to 30 l was added 1.5 ml of the 10-fold concentrated micro nutrient solution but it was used 0.58 g / l $Co(NO_3)_2$ instead of $CoCl_2$) in a photobioreactor at 48 °C. The harvested cells were stored at -80 °C. Membrane protein extraction was done with the following changes: all steps were conducted under dim green light. After cells were digested with lysozyme, cells were disrupted with a Yeda press at 3 MPa.[7] Thylakoids were prepared in buffer MCM (20 mM 2-(N-morpholino)-ethanesulfonic acid (MES-NAOH)–NaOH, pH 6; 20 mM $CaCl_2$; 10 mM $MgCl_2$). To remove phycobilisomes the thylakoid membranes were washed two times with high salt buffer MC-MCM (0.3 M $CaCl_2$, 0.15 M $MgCl_2$ in MCM buffer). Solubilization was done at a Chlorophyll a (Chla) concentration of 1.7 mM with 0.25 mM Pefabloc® SC-Protease Inhibitor (4-(2-aminoethyl)-benzylsulphonyl fluoride hydrochloride) for 5 min at room temperature; for n-dodecyl-ß-maltoside (DDM) in modified buffer MGCM (pH 6 instead of pH 6.5; 25 % glycerol (w/v) in MCM buffer) using 0.55 % (w/v) DDM and for octaethylene glycol monododecyl ether ($C_{12}E_8$) in buffer MBCM (0.5 M betaine monohydrate in MCM buffer) using 2.4 % $C_{12}E_8$. Before centrifugation for 30 min at 48000 rpm and 4 °C in a 50.2 Ti rotor (Beckman Instruments, USA) the mixture was diluted with the same volume of buffer; for DDM with a buffer containing 20 mM MES–NaOH, pH 6.0; 20 mM $CaCl_2$; 5 % (w/v) glycerol and for $C_{12}E_8$ with a buffer containing 20 mM MES–NaOH, pH 6.0; 10 mM $CaCl_2$; 0.5 M betaine monohydrate. The resulting supernatant was pooled and applied to the column.

For protein chromatography of DDM proteins buffers A (20 mM MES–NaOH, pH 6.0; 20 mM $CaCl_2$; 5 % (w/v) glycerol; 0.02 % DDM (v/v)), B (buffer A with 100 mM $MgSO_4$), A^- (buffer A without $CaCl_2$), C (5 mM MES–NaOH, pH 6.0; 60 mM $MgSO_4$; 0.02 % DDM (v/v)) and D (5 mM MES–NaOH, pH 6.0; 150 mM $MgSO_4$; 0.02 % DDM (v/v)) were used. In a first purification step DDM PSII and DDM PSI were separated. For this a column (50 mm in diameter and 380 mm in length, packed with Toyopearl DEAE-650 S) was equilibrated with 7 % buffer B in buffer A. After sample loading the column was washed first with 1 column volume (CV) with 7 % buffer B and subsequently with 1 CV 7.5 % buffer B. Protein elution was performed with linear gradients (from 7.5 % to 30 % buffer B in 1 CV, from 30 % to 33 % buffer B in 0.5 CV, from 33 % to 44 % buffer B in 0.1 CV and from 44 % to 70 % buffer B in

2.5 CV). DDM PSII eluted between 30 % and 33 % buffer B, DDM PSI between 50 % and 60 %.

DDM PSII containing fractions were pooled, diluted with approximately half the volume of buffer A^- and loaded onto a second column (35 mm in diameter and 470 mm in length, packed with Toyopearl DEAE-650 S) to separate the monomeric DDM PSII from dimeric. This column was equilibrated with 7 % buffer B in buffer A as well. After sample loading the column was first washed with 1.5 CV 7 % buffer B and then with 1.5 CV 7.5 % buffer B. Protein elution was performed with a linear gradient from 7 % to 22 % buffer B in 4.5 CV, including a holding step at 11 % buffer B for complete elution of the monomeric form. DDM PSII containing fractions were pooled, concentrated and washed in a modified buffer E (100 mM piperazine-N,N′-bis(2-ethanesulfonic acid) (PIPES)–NaOH, pH 7.0; 5 mM $CaCl_2$; 5 % glycerol; 0.03 % (v/v) DDM).

DDM PSI containing fractions from the first column were pooled and loaded onto a second column (50 mm in diameter and 130 mm in length, packed with Q Sepharose™ Fast Flow) equilibrated with buffer C. After column wash for 3 CV with buffer C the elution was performed with two linear gradients (from 10 % to 30 % buffer D in 1 CV and from 30 % to 100 % buffer D in 3 CV). DDM PSI containing fractions were pooled and concentrated.

Further purification of DDM PSI and DDM PSII was achieved by a pre-crystallization step.

For DDM PSI the concentrated sample was pre-crystallized through slow addition of buffer F (containing 5 mM MES–NaOH, pH 6.0 and 0.02 % DDM (v/v)) to a final conductivity of 0.5 – 0.7 mS/cm and kept at 4 °C for several days.

For DDM PSII the protein solution was adjusted to a Chla concentration of 0.74 mM in buffer E and mixed with the same volume of 12 % PEG2000 in 100 mM PIPES–NaOH, pH 7.0, 5 mM $CaCl_2$, 5 % glycerol. After incubation at 4 °C in the dark overnight, the solution was centrifuged and the pellet was redissolved, washed in buffer E and adjusted to a Chla concentration of 4 mM.

For protein chromatography of $C_{12}E_8$ proteins buffers G (20 mM MES–NaOH, pH 6.0; 10 mM $CaCl_2$; 0.5 M betaine monohydrate; 0.013 % $C_{12}E_8$), H (buffer G with 100 mM $MgSO_4$), I (40 mM MES-NaOH, pH 6.0; 5 mM $CaCl_2$; 0.5 M betaine monohydrate; 0.013 % $C_{12}E_8$), J (buffer I with 1 M NaCl), K (20 mM MES-NaOH, pH 5.0; 0.013 % $C_{12}E_8$), L (5 mM MES-NaOH, pH 5.5; 0.013 % $C_{12}E_8$) and M (buffer L with 0.5 M NaCl) were used. Similar to DDM proteins $C_{12}E_8$ PSII and $C_{12}E_8$ PSI were separated in a first purification step. For this, a column (50 mm in diameter and 380 mm in length, packed with Toyopearl DEAE-650 S) was equilibrated with 27 % buffer H in buffer G. After sample loading the column was washed with

27 % buffer H for 3 CV. Protein elution was performed with two linear gradients (from 27 % to 44.5 % buffer H in 0.7 CV and from 44.5 % to 47 % buffer G in 2 CV) followed by an isocratic step to 70 % buffer H. $C_{12}E_8$ PSII eluted between 37 % and 45 % buffer H, $C_{12}E_8$ PSI at 70 %.

$C_{12}E_8$ PSII containing fractions were pooled, diluted with approximately 2/3 the volume of buffer G and loaded onto a second column (35 mm in diameter and 470 mm in length, packed with Toyopearl DEAE-650 S) for further purification of $C_{12}E_8$ PSII. This column was equilibrated with 29 % buffer H in buffer G. After sample loading, the column was washed in 4 CV with stepwise increasing buffer H up to 31.5 %. Protein elution was performed with two linear gradients from 31.5 % to 50 % buffer H in 0.2 CV and from 50 % to 52 % buffer H in 2 CV. $C_{12}E_8$ PSII containing fractions were pooled and applied onto a third column (Mono Q™ 10/100 GL) equilibrated with 6 % buffer J in buffer I. Elution of $C_{12}E_8$ PSII was performed with a series of linear gradients and isocratic elution steps from 6 % to 25.5 % buffer J in a total volume of 21 CV. The $C_{12}E_8$ PSII containing fractions were pooled, concentrated and washed in modified buffer G (with 0.02 % $C_{12}E_8$) and adjusted to 2 mM Chla.

$C_{12}E_8$ PSI containing fractions from the first column were pooled and mixed with approximately one volume of buffer K to adjust the pH to 5.5 and the conductivity to 3-4 mS/cm. This mixture was loaded on a column (35 mm in diameter and 310 mm in length, packed with SP Sepharose™ Fast Flow) pre-equilibrated with 10 % buffer M in buffer L. After column wash for 2 CV with 10 % buffer M the elution was performed with a linear gradient (from 10 % to 35 % buffer M in 1.5 CV) and an isocratic step at 35 %. $C_{12}E_8$ PSI containing fractions were pooled, concentrated and washed in a buffer containing 5 mM MES-NaOH, pH 6; 30 mM $MgSO_4$; 0.013 % $C_{12}E_8$.

Further purification of $C_{12}E_8$ PSI was achieved by a pre-crystallization step, where the concentrated sample was adjusted to a Chla concentration of 10 mM and mixed 1:50 with a buffer containing 5mM MES-NaOH, pH 6; 0.013% $C_{12}E_8$. This mixture was kept at 4 °C for several days.

Sample analysis

All sample analysis was done with pre-crystallized photosystem dissolved in buffer. Blue native- (BN) and sodium dodecyl sulfate- (SDS) polyacrylamide gel electrophoresis (PAGE) gels were run (see Figure S2), to ensure the purity and the subunit composition of the samples. The 1.5 mm BN-PAGE gels consisted of four layers of 4 - 8.5 % acrylamide concentration.

The samples were run at 1 µg (PSII) or 2 µg (PSI) of Chla at 12 mA per gel with anode (25 mM Imidazole, pH 7) and cathode buffer (50 mM Tricine, 7.5 mM Imidazole, 0.02 % Coomassie G-250, pH 7), the latter was exchanged to 1/10 cathode buffer (50 mM Tricine, 7.5 mM Imidazole, 0.002 % Coomassie G-250, pH 7) after ~1/3 of the sample run was completed. Subsequently, the gels were destained over night in destaining solution (10 % acetic acid).
SDS-PAGE was done after Laemmli[8] using a 5 % acrylamide stacking gel (124.8 mM Tris-HCl at pH 6.8, 0.1 % SDS) and the separating gel consisted of two layers of 12 % and 15 % acrylamide for PSI and only 12 % for PSII (375 mM Tris-HCl pH 8.8, 0.1 % SDS). After mixing the photosystems with SDS sample buffer (62.5 % Tris-HCl pH 6.5; 100 mM Dithiothreitol (DTT); 10 % glycerol; 0.1 % Bromphenol blue; 2 % (w/v) SDS), the PSI (5 µg Chla) was denatured for 1 h at 60 °C and the PSII (2 µg of Chla) for 10 min at 60 °C. The SDS-gels were run at room temperature (RT) at 20 mA / gel in SDS running buffer (25 mM Tris, 192 mM glycine, 0.1 % SDS) and kept in staining solution (7.8 % (w/v) ammonium sulfate, 1.57 % (w/v) orthophosphoric acid, 0.1 % Coomassie G-250, 4.9 % methanol) at RT over night. Finally, the SDS gels were destained for 1 h at RT.
The biggest subunits of PSI (PsaA and PsaB) exhibit a molecular weight of about 83 kDa but run in the SDS gel at around 60 kDa.
In addition, we did MALDI-ToF analysis of the samples up to the size of 20 kDa in linear positive-ion mode by means of a MicroFlex MALDI-ToF mass spectrometer (Bruker Daltonics Ultraflex II, FU Berlin) using 500/500 shots with a frequency of 2000/s and data were analysed with fleXcontrol (Bruker Daltonics). The PS samples were denatured 1:30 in 0.1 % (v/v) Trifluoro acetic acid (TFA) and mixed 1:1 on the target plate with the matrix solution (sinapinic acid in 40% acetonitrile, 0.1% TFA).
Furthermore, size exclusion chromatography of the photosystem samples was performed with a Superose™ 6 Prep Grade (17-0489-01, GE Healthcare Bio-Sciences, Uppsala) column (d = 1 cm, h = 30 cm, CV = 23.56 ml) at a constant flow rate of 0.5 ml / min. The column was equilibrated (3-5 CV) and eluted (2-3 CV) with the respective buffer (G60 for DDM PSI, storage buffer for $C_{12}E_8$ PSI, 100 mM PIPES pH 7.0; 5 % glycerin; 5mM calcium chloride; 0.03 % DDM for DDM PSII and 20 mM MES pH 6.0; 0.5 M betaine monohydrate; 10 mM calcium chloride; 0.013 % $C_{12}E_8$ for $C_{12}E_8$ PSII) and each sample dissolved in the respective buffer was filtered trough a 0.2 µm filter (Puradisc 30/0.2 CA S, Whatman). Afterwards the 0.5 ml of each sample (capillary loop emptied with 1 ml of respective equilibration buffer) were loaded onto the column and elution showed a faster passing trough the column for the $C_{12}E_8$ samples (see Figure S4) compared to samples in DDM buffer.

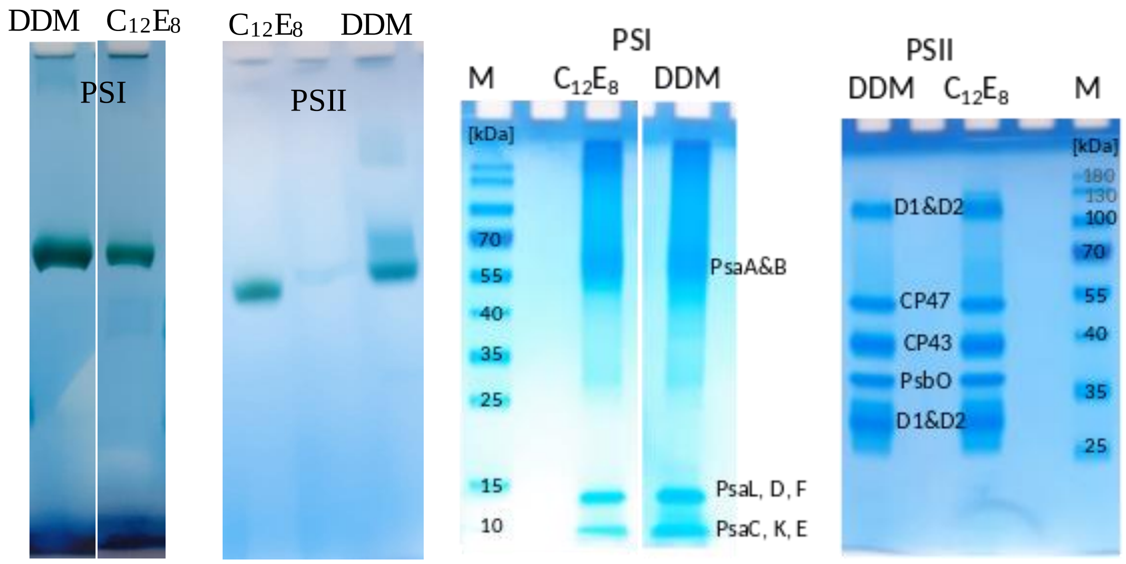

Figure S2: BN (left images) and SDS (right images) gels of PSI and PSII. The protein bands in the BN gels indicating a high purity and SDS gels in PSI reveal the presence of the big subunits (PsaA and PsaB) as well as bands around 15 kDa and 10 kDa corresponding to PsaL, D, F and PsaC, K, E, respectively. For PSII the subunits CP47 (PsbB), CP43 (PsbC), PsbO, D1 (PsbA) and D2 (PsbD) can be detected via SDS; upper band of D1&D2 are probably not fully digested.

Table S2: MALDI-ToF data of PSI without PsaA&B (already shown in SDS-PAGE gels; $C_{12}E_8$ PSI not shown) and PSII in comparison to the PhD thesis of J. Kern[9]; the subunits PsbA-D and PsbO are already shown in SDS-PAGE gels.

**Kern measured $\bar{x} \pm \sigma$** (*calculated*) [m/z]	**PSII subunits**	**DDM PSII measured** $\bar{x} \pm \sigma$ [m/z] (n=9)	**C12E8 PSII measured** $\bar{x} \pm \sigma$ [m/z] (n≥3)	**PSI subunits**	**DDM PSI measured** $\bar{x} \pm \sigma$ [m/z] (n=13)
3906 ± 4	T	3902 ± 1	3903 ± 1	L (z=2)	16121 ± 5 (8070±4)
4011 ± 2	M	3917 ± 1	3918 ± 1	D	15241 ± 3
4017 ± 4	J	4015 ± 1	4016 ± 1	F	15126 ± 6
4103 ± 4	K	4099 ± 1	4100 ± 1	C	8670 ± 1
4192 ± 4	X	4187 ± 1	4189 ± 1	K	8390 ± 2
4301 ± 4	L	4296 ± 1	4297 ± 1	E	8259 ± 2
4437 ± 4	I	4433 ± 1	4434 ± 1	J	4796 ± 1
4617 ± 4	Y	4613 ± 1	4614 ± 0	I	4144 ± 29
4981 ± 5	F	4976 ± 1	4977 ± 1	X	3973 ± 4
6798 ± 5	Z	6794 ± 1	6793 ± 0	M	3424 ± 1
7227 ± 5	H	7179 ± 1	7181 ± 2		
(7875)	V, z=2	7873 ± 1	7874±0		
9446 ± 6	E	9442 ± 1	9442 ± 1		
11649 ± 8	U	11644 ± 1	11644 ± 4		
15752 ± 11	V	15744 ± 1	15745 ± 7		
15752 ± 11	V	15744 ± 1	15745 ± 7		

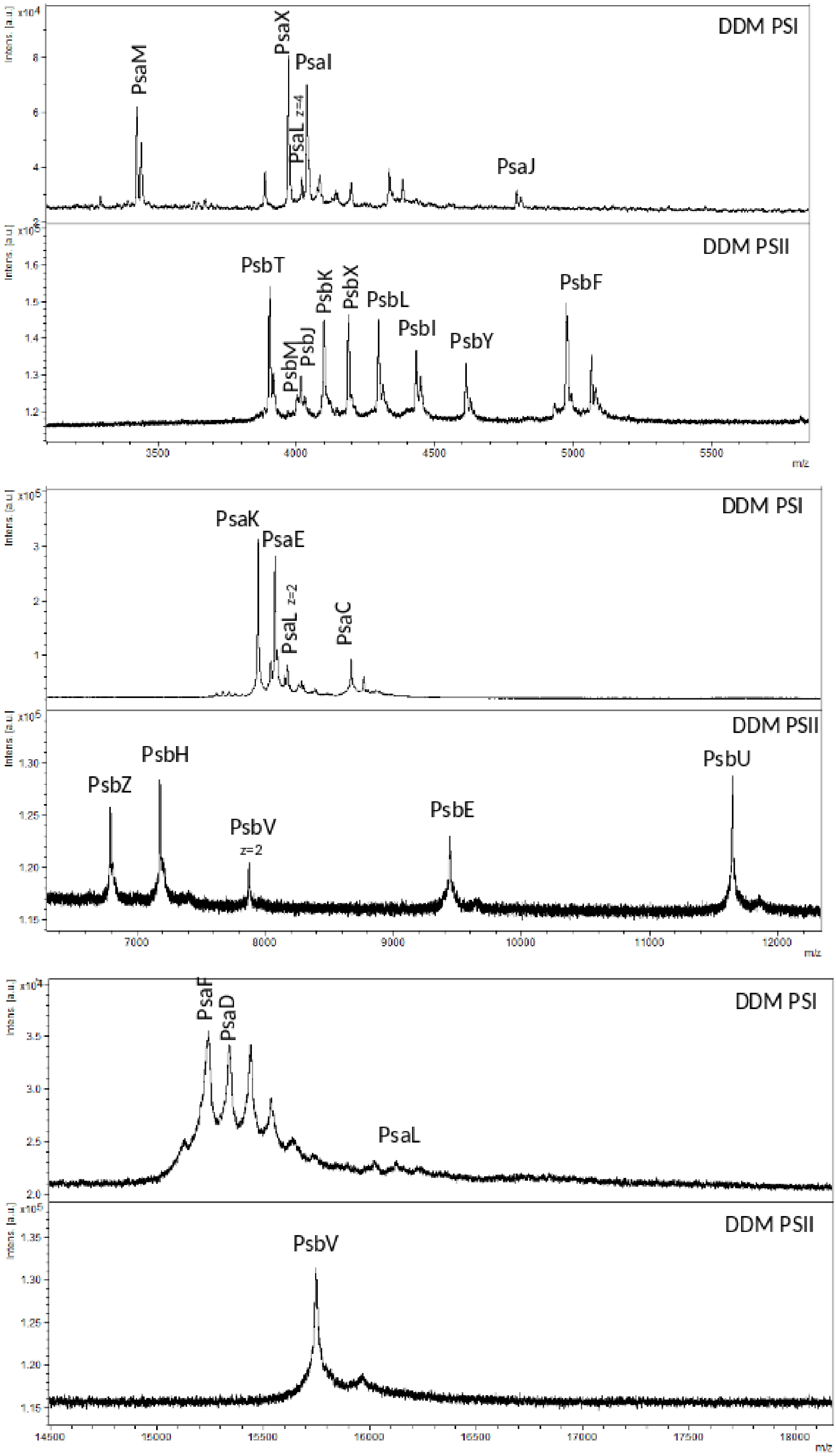

Figure S3: Exemplary MALDI-ToF spectra of DDM PSI (upper curve) and PSII (lower curve) from 3100-18100 m/z.

Altogether, the analysis of our photosystems in different detergents show the consistency of our purification procedure resulting in intact proteins without loss of subunits.

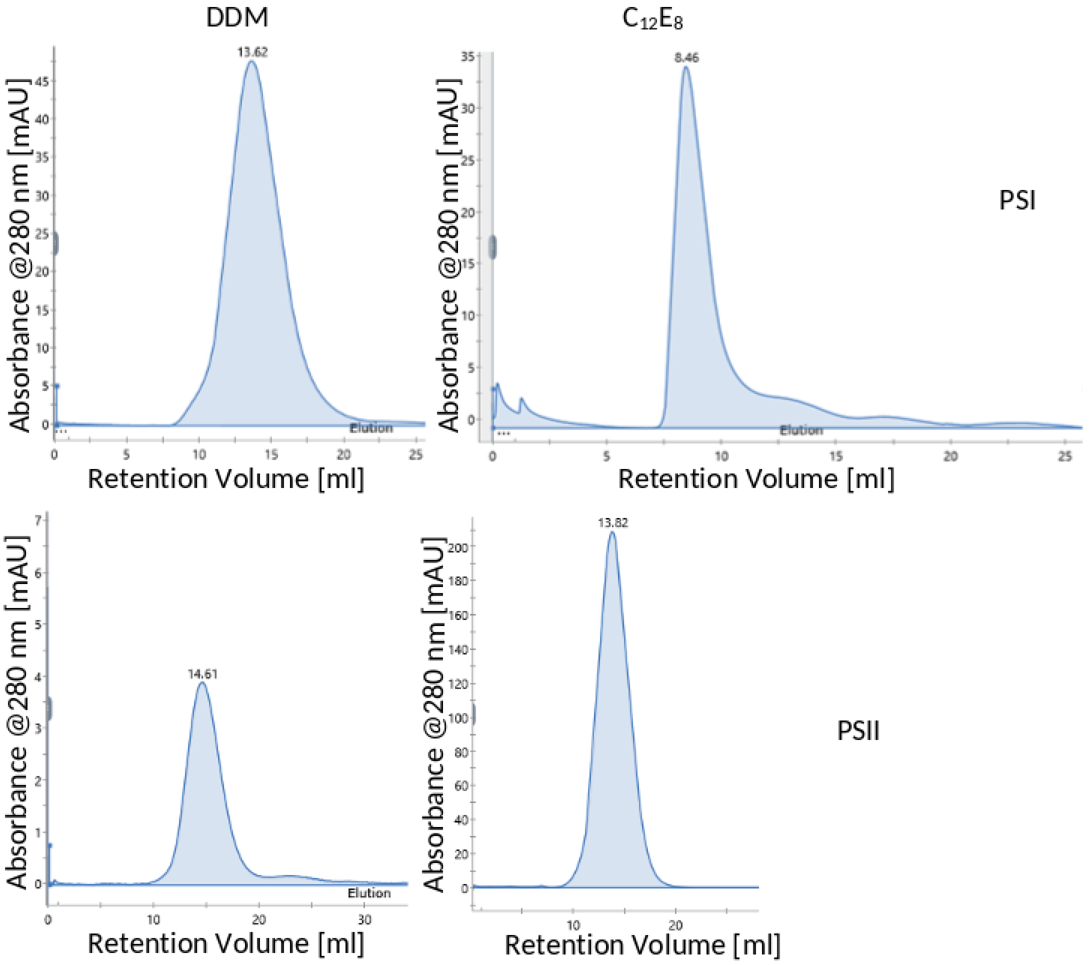

Figure S4: Exemplary size exclusion chromatograms of dissolved photosystems after pre-crystallization, PSI (top row) and PSII (lower row) purified in either DDM (left) or $C_{12}E_8$ (right), each n = 3. Be aware of the different concentration of the shown filtered photosystem samples and the varying scaling on both axis of each graph. Apparently, the $C_{12}E_8$ buffer results in a faster elution time for both proteins.

The shown chromatograms (see Figure S4) depict PSI and PSII samples at different concentrations (y-axis scaling varies for each graph) and the small signals for DDM PSII and $C_{12}E_8$ PSI after the main peak are in the range of the recorded disturbance from the injection of the sample in the beginning of each elution and can therefore be attributed to the column run.

Activity measurements

The oxygen (O_2) activity of the samples was assessed at 25 °C (for PSII) or at 20 °C (for PSI) via a Clarke-type electrode (Oxygraph+, Hansatech, Germany) under constant mixing at 60 rpm and excitation by illumination of > 500 $\mu mol_{photons}$ / (m^2 • s) in triplicate.

The DDM PSII and $C_{12}E_8$ PSII samples showed O_2 evolution rates between 1100 and 1600 µmol O_2 / (mg Chla • h), as measured in buffer containing 20 mM MES–NaOH, pH 6.0,

20 mM $CaCl_2$, 5% (w/v) glycerol, and 1 mM 2,5-dichloro-p-benzoqinone as artificial electron acceptor.

The O_2 consumption rates for DDM PSI and $C_{12}E_8$ PSI samples were measured between -1662 and -2150 µmol O_2 / (mg Chla • h) in buffer containing 25 mM tricine-NaOH, pH 8.0, 300 µM methyl viologen, 1 mM sodium ascorbate, 16 µM cyt c_{HH} and either 0.013 % $C_{12}E_8$ or 0.02 % DDM. Prior to measurement, PSI crystals were redissolved in a buffer containing 25 mM tricine-NaOH, pH 8, 200 mM $MgSO_4$ and 0.02 % DDM or 5 mM MES-NaOH, pH 6, 30 mM $MgSO_4$, and 0.013 % $C_{12}E_8$.

Dynamic light scattering (DLS)

DLS measurements were done via a DynaPro NanoStar (Wyatt technology) at 787 nm and 20 °C in buffer (for PSI: 25 mM Tricine-NaOH, pH 8; 50 mM NaCl and either 0.013 % $C_{12}E_8$ or 0.02 % DDM; for PSII: 100 mM Pipes, pH 7; 5 mM $CaCl_2$; 5 % glycerol; 0.03 % DDM or 20 mM MES-NaOH, pH 6.0; 20 mM $CaCl_2$; 0.5 M betaine 0.013 % $C_{12}E_8$) in 4 µl-disposable MicroCuvettes (Wyatt technology) after filtering of each sample (22 µm syringe driven filter unit, Millex® - GV). All used DLS measurements (n ≥ 3) show Pd-values below 15 % indicating a homogeneous solution.

Detergent determination

To determine the detergent belt, samples with constant Chla concentration but different detergent concentrations were measured. Determination of detergent concentration was done based on the method of DaCosta and Baenziger (2003) through Fourier Transform Infrared Spectroscopy (FTIR) with the Direct Detect® Spectrometer from Merck KGaA at 2840-70/cm, with absorbance set to zero at 2600 / cm.

To reduce the detergent concentration, we assumed that the storage detergent concentration (DDM PSI: 0.02 % DDM, $C_{12}E_8$ PSI: 0.013 % $C_{12}E_8$, DDM PSII: 0.03 % DDM, $C_{12}E_8$ PSII: 0.02 % $C_{12}E_8$) is our starting concentration. The samples were mixed with the respective detergent-free buffer to reach a certain detergent concentration and washed four times with the respective detergent-containing buffer.

In Figure S5 are shown the calibration curves for $C_{12}E_8$ (left) and DDM (right). The PDC FTIR signal can be seen in Figure S6 where the protein (at 2870 / cm) and PDC (at 2855 / cm) peak are used to calculate the detergent concentration.

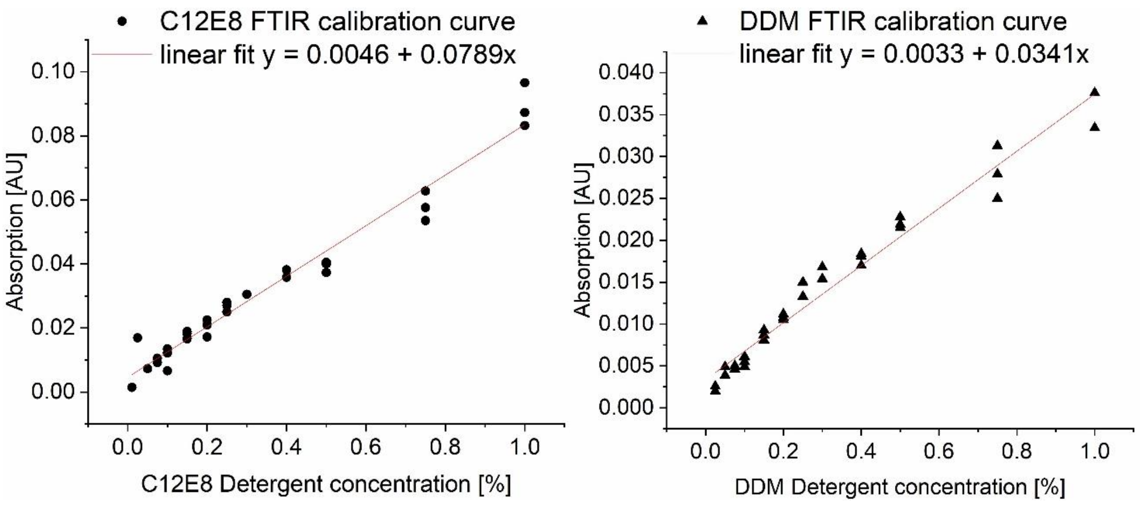

Figure S5 : Calibration curves for determining the detergent concentration via FTIR for $C_{12}E_8$ and DDM.

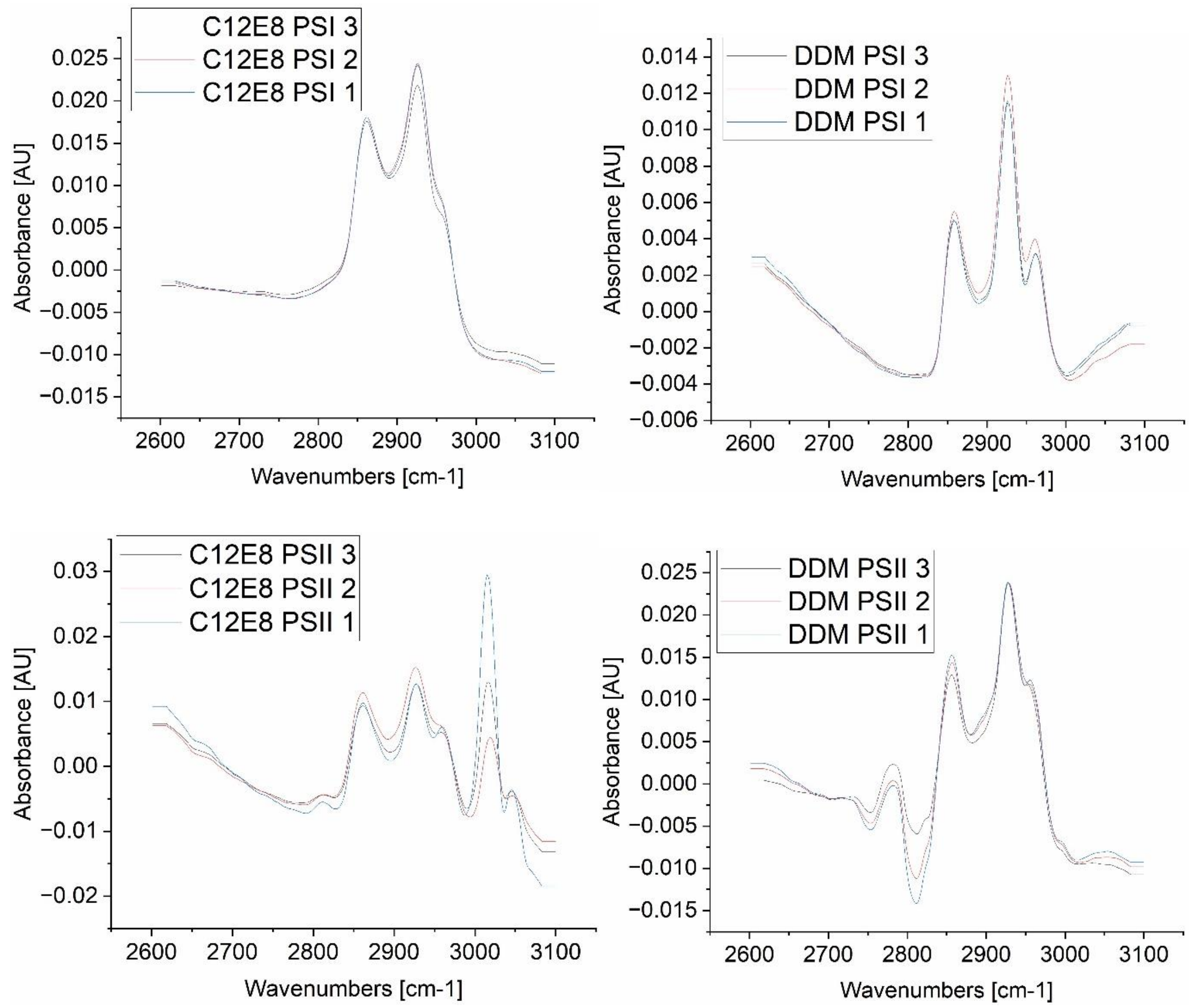

Figure S6: FTIR spectra of $C_{12}E_8$ PSI and PSII (left) as well as DDM PSI and PSII (right).

While getting approximate detergent concentration values for detergent in buffer, the results for the PDC solution could differ. Another method is determining the detergent concentration

successfully by using MALDI-ToF[10] and could be used in the future to determine the detergent concentration in the PDC solution exactly. However, since these experiments are not performed in-house during the detergent reduction phase of the sample preparation, the detergent determination via MALDI-ToF could maybe only find application after this process.

SAXS measurements of the buffers show the increase of the intensity with rising detergent concentrations (see Figure S7).

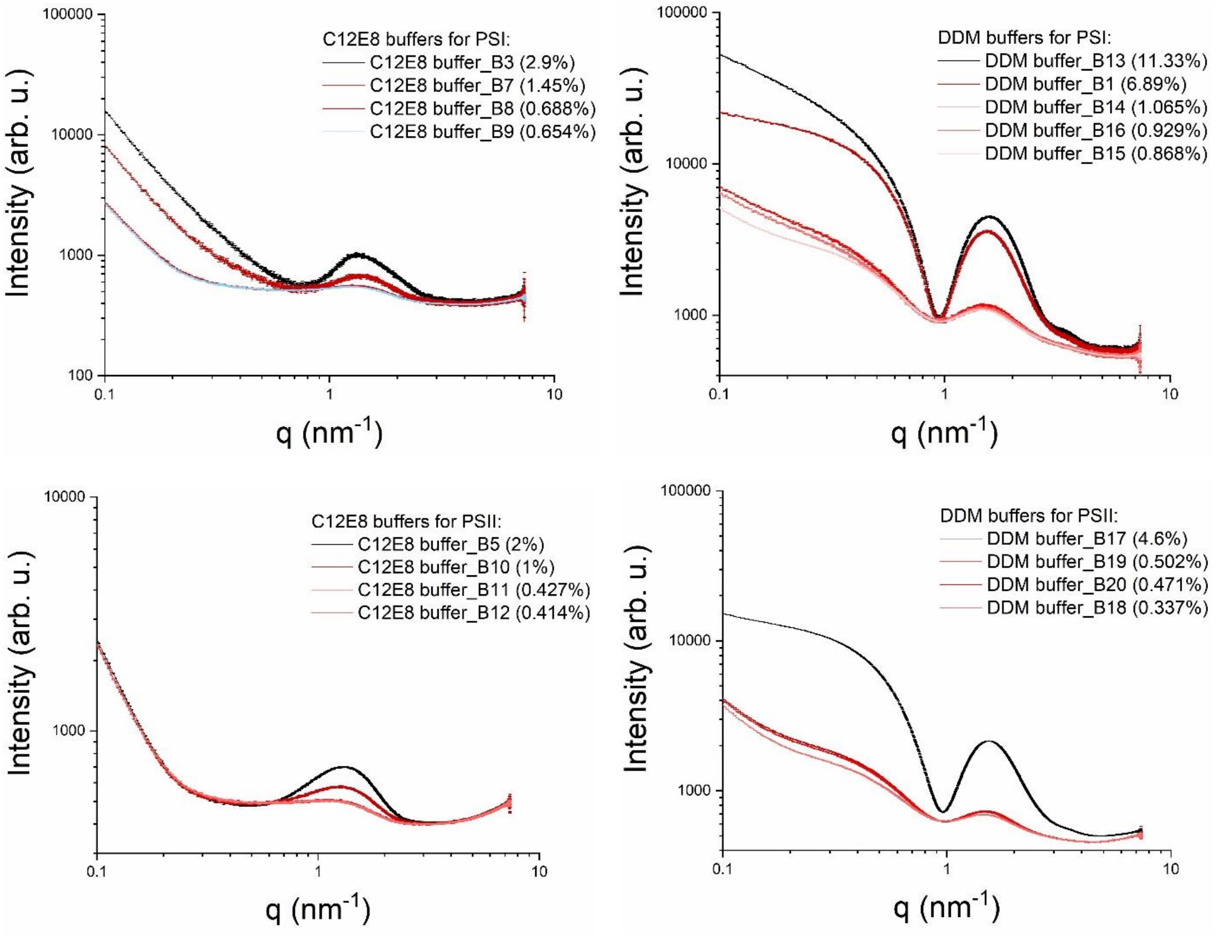

Figure S7: SAXS buffer measurements without protein with varying detergent concentration ($C_{12}E_8$ on the left and DDM on the right) determined via FTIR previously.

Figure S7 illustrates the SAXS curves obtained from a concentration series of DDM and $C_{12}E_8$ buffer solutions. The data reveal a noticeable variation in the scattering profiles as a function of detergent concentration in the buffer. Specifically, higher detergent concentrations (for both DDM and $C_{12}E_8$) result in an increase in peak intensity at a q-value of 1.1 nm^{-1}, corresponding to the concentration of DDM or $C_{12}E_8$ micelles. Due to the unavailability of the SEC-SAXS

setup caused by technical issues, identifying an appropriate buffer solution was critical for accurate buffer subtraction and to avoid over-subtraction.

Buffer subtraction examples for PSII and PSI in solution with $C_{12}E_8$ detergent

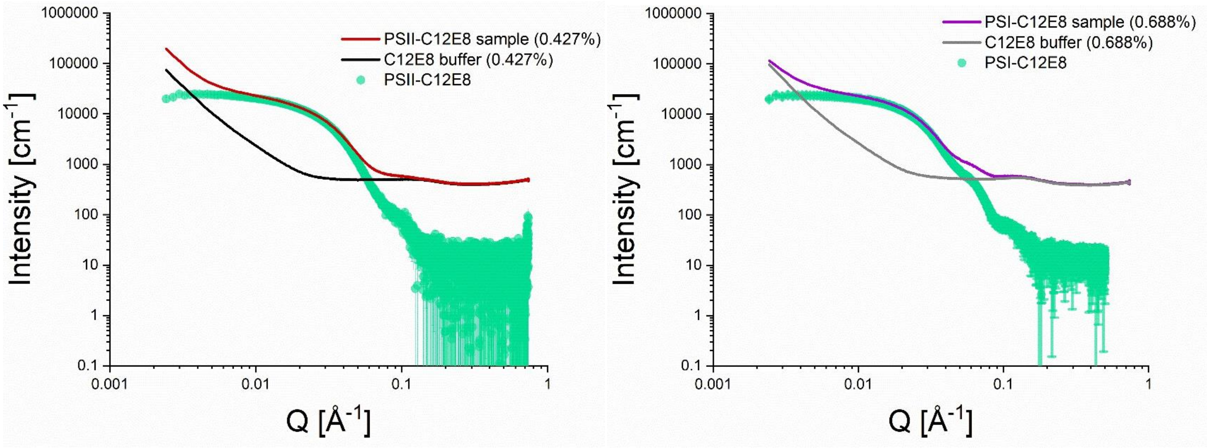

Figure S8: Buffer subtraction for PSII (left) and PSI (right) samples in solution with $C_{12}E_8$ detergent.

In contrast to DDM, the $C_{12}E_8$ curves show no additional peak after buffer subtraction (see Figure S8).

Determination of detergent belt

Recently, a Cryo-EM structure of DDM PSII was published at a resolution of 1.71 Å (pdb 9evx, map emd_50019).[3] Here we calculated the volume and size dimensions of the DDM belt around the 9evx DDM PSII structure by using the ChimeraX software (version 1.7.1)[11–13] as described before[14]. We determined the length of the PSII PDC to D_{max} = 238 Å (D_{min} = 156 Å) as well as the volume of the DDM detergent belt around the PSII to 492.5 Å^3 (see Figure S9) by using the volume of the whole PDC at a contour level of 0.0718 and the volume of the PSII without detergent belt at a contour level of 0.246. Furthermore, we found the thickness of the detergent belt to be in-between 30 – 55 Å by utilising the ChimeraX measuring tool at a contour level of 0.0718.

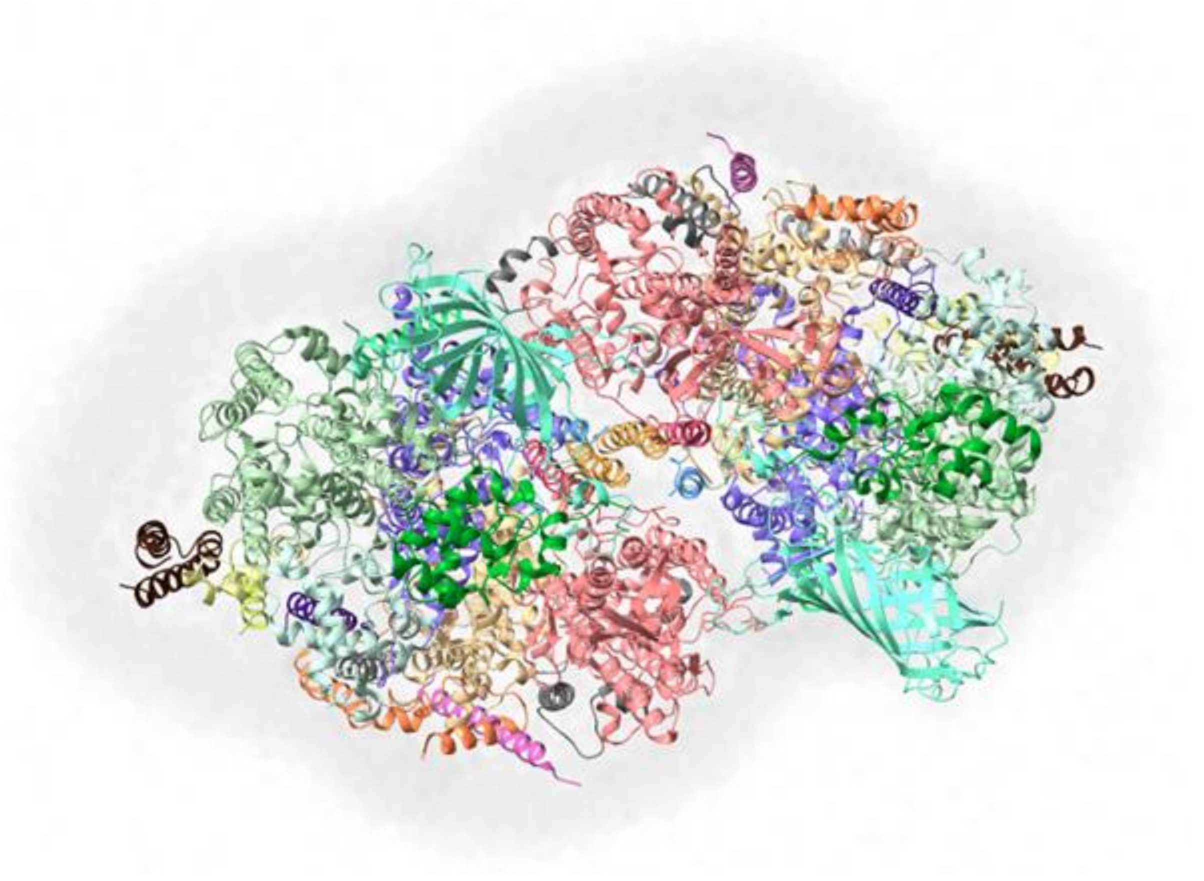

Figure S9: Top view of Cryo-EM DDM PSII pdb 9evx[3] with visible detergent belt.

References

1. Loll, B.; Saenger, W. Towards complete cofactor arrangement in the 3.0 Å resolution structure of photosystem II. *Nature* **2005** (438), 1040–1044.

2. Hussein, R.; Ibrahim, M.; Bhowmick, A.; Simon, P. S.; Chatterjee, R.; Lassalle, L.; Doyle, M.; Bogacz, I.; Kim, I.-S.; Cheah, M. H.et al. Structural dynamics in the water and proton channels of photosystem II during the S2 to S3 transition. *Nat. Comm.* **2021,** *12* (1), 6531. DOI: 10.1038/s41467-021-26781-z.

3. Hussein, R.; Graça, A.; Forsman, J.; Aydin, A. O.; Hall, M.; Gaetcke, J.; Chernev, P.; Wendler, P.; Dobbek, H.; Messinger, J. et al. Cryo-electron microscopy reveals hydrogen positions and water networks in photosystem II. *Science (New York, N.Y.)* **2024,** *384* (6702), 1349–1355. DOI: 10.1126/science.adn6541.

4. Sakurai, I.; Shen, J.-R.; Leng, J.; Ohashi, S.; Kobayashi, M.; Wada, H. Lipids in oxygen-evolving photosystem II complexes of cyanobacteria and higher plants. *Journal of biochemistry* **2006,** *140* (2), 201–209. DOI: 10.1093/jb/mvj141.

5. Kern, J.; Loll, B.; Lüneberg, C.; DiFiore, D.; Biesiadka, J.; Irrgang, K.-D.; Zouni, A. Purification, characterisation and crystallisation of photosystem II from Thermosynechococcus elongatus cultivated in a new type of photobioreactor. *Biochimica et Biophysica Acta* **2005,** *1706* (1-2), 147–157. DOI: 10.1016/j.bbabio.2004.10.007.

6. Castenholz, R. W. [3] Culturing methods for cyanobacteria. In: *Cyanobacteria*; Methods in Enzymology; Elsevier, 1988, pp 68–93. DOI: 10.1016/0076-6879(88)67006-6.

7. Schatz, G. H.; Witt, H. T. Extraction and characterization of oxygen-evolving Photosystem II complexes from a thermophilic cyanobacterium Synechococcus spec. *Photobiochemistry and Photobiophysics* **1984,** *7* (1), 1–14. DOI: 10.1016/S0165-8646(24)00613-5.

8. Laemmli, U. K. Cleavage of structural proteins during the assembly of the head of bacteriophage T4. *Nature* **1970,** *227* (5259), 680–685. DOI: 10.1038/227680a0.

9. Kern, J. Structural and functional investigations of Photosystem II from Thermosynechococcus elongatus. PhD thesis, Technische Universität Berlin, Berlin, 2005. https://depositonce.tu-berlin.de/items/cd5b7acb-d5b5-47a0-9c0c-a6af28d0d03a (accessed 02.06.25).

10. Chaptal, V.; Delolme, F.; Kilburg, A.; Magnard, S.; Montigny, C.; Picard, M.; Prier, C.; Monticelli, L.; Bornert, O.; Agez, M. et al. Quantification of Detergents Complexed with Membrane Proteins. *Scientific Reports* **2017,** *7,* 41751, 1-12. DOI: 10.1038/srep41751.

11. Goddard, T. D.; Huang, C. C.; Meng, E. C.; Pettersen, E. F.; Couch, G. S.; Morris, J. H.; Ferrin, T. E. UCSF ChimeraX: Meeting modern challenges in visualization and analysis. *Protein Science : a Publication of the Protein Society* **2018,** *27* (1), 14–25. DOI: 10.1002/pro.3235.

12. Meng, E. C.; Goddard, T. D.; Pettersen, E. F.; Couch, G. S.; Pearson, Z. J.; Morris, J. H.; Ferrin, T. E. UCSF ChimeraX: Tools for structure building and analysis. *Protein Science : a Publication of the Protein Society* **2023,** *32* (11), e4792, 1-13. DOI: 10.1002/pro.4792.

13. Pettersen, E. F.; Goddard, T. D.; Huang, C. C.; Meng, E. C.; Couch, G. S.; Croll, T. I.; Morris, J. H.; Ferrin, T. E. UCSF ChimeraX: Structure visualization for researchers, educators, and developers. *Protein Science : a Publication of the Protein Society* **2021,** *30* (1), 70–82. DOI: 10.1002/pro.3943.

14. Zampieri, V.; Gobet, A.; Robert, X.; Falson, P.; Chaptal, V. CryoEM reconstructions of membrane proteins solved in several amphipathic solvents, nanodisc, amphipol and detergents, yield amphipathic belts of similar sizes corresponding to a common ordered solvent layer. *Biochimica et Biophysica Acta. Biomembranes* **2021,** *1863* (11), 183693; 1-9. DOI: 10.1016/j.bbamem.2021.183693.
